# Exploring the relationship between ultra-processed food consumption and gut microbiota at school age in a Brazilian birth cohort

**DOI:** 10.1590/0102-311XEN094424

**Published:** 2025-03-31

**Authors:** Etiene Dias Alves, Marina Xavier Carpena, Aluísio J. D. Barros, Elena M. Comelli, Lorena López-Domínguez, Robert H. J. Bandsma, Iná da Silva dos Santos, Alicia Matijasevich, Juliana dos Santos Vaz, Romina Buffarini, Isabel Oliveira Bierhals, Maria Carolina Borges, Luciana Tovo-Rodrigues

**Affiliations:** 1 Universidade Federal de Pelotas, Pelotas, Brasil.; 2 Universidade Federal de Rio Grande, Rio Grande, Brasil.; 3 Faculdade de Medicina, Universidade Federal de Pelotas, Pelotas, Brasil.; 4 Temerty Faculty of Medicine, University of Toronto, Toronto, Canada.; 5 Translational Medicine Program, Hospital for Sick Children, Toronto, Canada.; 6 Faculdade de Medicina, Universidade de São Paulo, São Paulo, Brasil.; 7 Faculdade de Nutrição, Universidade Federal de Pelotas, Pelotas, Brasil.

**Keywords:** Gut Microbiota, Ultra-Processed Food, Adolescence, Cohort Studies, Actinobacteria, Microbiota Intestinal, Alimentos Ultraprocessados, Adolescência, Estudos de Coortes, Actinobacteria, Microbiota del Intestino, Alimentos Ultraprocesados, Adolescencia, Estudios de Cohortes, Actinobacteria

## Abstract

Dietary patterns significantly impact health outcomes and gut microbiota composition. However, longitudinal studies associating ultra-processed food consumption with gut microbiota composition, especially among adolescents in low- and middle-income countries, are lacking. This study aimed to explore this association using data collected from 364 participants at ages 6, 11, and 12 years from the 2004 Pelotas (Brazil) Birth Cohort. Microbiota data was obtained at age 12 after 16S rRNA gene sequencing of self-collected fecal samples. Linear or logistic regression models evaluated the relationship between age groups and gut microbiota outcomes (alpha diversity, beta diversity and relative abundances at the phylum and genus levels), considering dietary covariates and demographic, socioeconomic, health-related, and behavioral factors. No significant associations between ultra-processed food consumption and alpha diversity were observed after multiple testing corrections, and there was no strong evidence linking ultra-processed food consumption and beta diversity, with unweighted metrics explaining little variance at ages 11 and 12. Nominal associations were found between ultra-processed food and relative abundances of *Actinobacteria* (p = 0.032) and *Proteobacteria* (p = 0.045) (phyla), *Bacteroides* (p = 0.037 at age 6; p = 0.015 at age 11) and *Peptostreptococcus* (p = 0.025 at age 6; p = 0.010 at age 11) (genera). However, these associations lost statistical significance after adjustments for multiple comparisons. These findings highlight the need for more longitudinal studies to better understand the complex interaction between ultra-processed food intake and gut microbiota composition in adolescent populations in low- and middle-income countries.

## Resumo

Os padrões alimentares impactam significativamente os desfechos de saúde e a composição da microbiota intestinal. No entanto, estudos longitudinais sobre o consumo de alimentos ultraprocessados e a composição da microbiota intestinal, especialmente entre adolescentes em países de baixa e média renda, são escassos. Este estudo teve como objetivo explorar essa associação utilizando dados coletados de 364 participantes aos 6, 11 e 12 anos, da Coorte de Nascimentos de Pelotas (Brasil) de 2004. Os dados sobre microbiota foram obtidos aos 12 anos por meio de sequenciamento do gene 16S rRNA de amostras fecais autocoletadas. Modelos de regressão linear e logística avaliaram a relação entre faixas etárias e resultados da microbiota intestinal (diversidade alfa, diversidade beta e abundâncias relativas nos níveis de filo e gênero), considerando fatores demográficos, socioeconômicos, relacionados à saúde, comportamentais e covariáveis dietéticas. Não foram observadas associações significativas entre o consumo de alimentos ultraprocessados e a diversidade alfa após correção para múltiplos testes, e não foram encontradas evidências de uma ligação entre o consumo de alimentos ultraprocessados e a diversidade beta, com as métricas não ponderadas explicando pouca variância aos 11 e 12 anos. Foram encontradas associações nominais entre o consumo de alimentos ultraprocessados e as abundâncias relativas de *Actinobacteria* (p = 0,032) e *Proteobacteria* (p = 0,045) (filos), assim como entre *Bacteroides* (p = 0,037 aos 6 anos; p = 0,015 aos 11 anos) e *Peptostreptococcus* (p = 0,025 aos 6 anos; p = 0,010 aos 11 anos) (gêneros). No entanto, essas associações perderam significância estatística após os ajustes para comparações múltiplas. Esses achados destacam a necessidade de mais estudos longitudinais para compreender melhor a complexa interação entre o consumo de alimentos ultraprocessados e a composição da microbiota intestinal em populações adolescentes de países de baixa e média renda.

## Resumen

Los patrones dietéticos afectan significativamente la salud y la composición de la microbiota intestinal. No obstante, se observan escasos estudios longitudinales sobre el consumo de alimentos ultraprocesados y la composición de la microbiota intestinal, especialmente entre adolescentes de países de ingresos bajos y medianos. Este estudio tuvo como objetivo explorar esta asociación a partir de datos recopilados de 364 participantes de 6, 11 y 12 años de edad de la Cohorte de Nacimientos de Pelotas (Brasil), 2004. Los datos de la microbiota se obtuvieron a los 12 años mediante la secuenciación del gen ARNr 16S de muestras fecales autorecogidas. Los modelos de regresión lineal y logística evaluaron la relación entre los grupos de edad y los resultados de la microbiota intestinal (diversidad alfa, diversidad beta y abundancias relativas a nivel de filo y género) teniendo en cuenta las covariables demográficas, socioeconómicas, relacionadas con la salud, el comportamiento y la dieta. No se observaron asociaciones significativas entre el consumo de alimentos ultraprocesados y la diversidad alfa después de la corrección para múltiples pruebas, y no se encontró evidencia de un vínculo entre el consumo de alimentos ultraprocesados y la diversidad beta, con métricas no ponderadas que explican poca varianza a los 11 y 12 años. Se encontraron asociaciones nominales entre el consumo de alimentos ultraprocesados y las abundancias relativas de *Actinobacteria* (p = 0,032) y *Proteobacteria* (p = 0,045) (filos), así como entre *Bacteroides* (p = 0,037 a los 6 años; p = 0,015 a los 11 años) y *Peptostreptococcus* (p = 0,025 a los 6 años; p = 0,010 a los 11 años) (géneros). Sin embargo, estas asociaciones perdieron significación estadística después de los ajustes para comparaciones múltiples. Estos hallazgos resaltan la necesidad de realizar más estudios longitudinales para conocer mejor la compleja interacción entre el consumo de alimentos ultraprocesados y la composición de la microbiota intestinal en poblaciones de adolescentes de países de ingresos bajos y medianos.

## Introduction

Habitual dietary intake is important to shape the unique and stable profile of an individual’s gut microbiome ^1^, influencing its abundance and diversity ^2^ ^,^ ^3^ ^,^ ^4^. The gut microbiota is pivotal in various physiological processes, including metabolism and immune function ^5^, and may play a role in the etiology of several diseases, such as obesity and type 2 diabetes mellitus ^6^ ^,^ ^7^.

Ultra-processed foods are products primarily derived from food substances and industrial ingredients, characterized as processed and packaged as ready-to-eat items ^8^. Its use has been linked to a number of health issues ^9^, including obesity, diabetes, and non-alcoholic fatty liver disease in both adults and children ^10^ ^,^ ^11^ ^,^ ^12^ ^,^ ^13^. Notably, ultra-processed food consumption is rising in middle- and high-income countries ^14^ ^,^ ^15^, attributed to their convenience and extended shelf life due to high levels of processing ^8^.

Adolescence is marked by profound changes in physical, mental, social, and lifestyle transformations ^16^. This stage presents a critical opportunity to promote and sustain health dietary practices ^17^, such as preventing ultra-processed food consumption, which is essential for short- and long-term health, in the light of the global obesity crisis ^17^. The composition of gut microbiota in adolescents and potential impact of ultra-processed food consumption on this microbiota are not well elucidated.

Investigation regarding gut microbiota and its association with ultra-processed foods largely targets adult populations from high-income countries, primarily using a cross-sectional design ^18^ ^,^ ^19^ ^,^ ^20^. Existing studies in adult cohorts have shown that ultra-processed food consumption correlates with alterations in gut microbiota composition ^21^. Evidence suggest that individuals with a higher ultra-processed food intake exhibit a less diverse microbiota, characterized by distinct variations in predominant phyla ^18^ ^,^ ^22^ ^,^ ^23^. For instance, at the genus level, a study in a sample of senior subjects revealed that higher ultra-processed food consumption was positively associated with relative abundance of *Alloprevotella*, *Negativibacillus*, *Prevotella* and *Sutterella* ^24^, corroborating the findings of two other studies with adult samples ^20^ ^,^ ^25^. The findings also revealed that diets high in energy density and low in fiber, often associated with ultra-processed food consumption, are linked to a microbiota profile with increased *Firmicutes* and reduced *Bacteroidetes* at the phylum level, correlating with a heightened risk of obesity ^26^.

Research involving healthy children and adolescents ^2^, particularly those from low- and middle-income countries, is essential to understand the effects of ultra-processed food consumption on gut microbiota across different life stages and in varied populations. Additionally, long-term consequences of ultra-processed food intake on gut microbiota composition remain uncertain. Therefore, this study aimed to explore the association of ultra-processed food consumption at three distinct moments (ages 6, 11, and 12) in relation to microbiota composition at age 12, in a subsample of participants from the 2004 Pelotas (Brazil) Birth Cohort.

## Methods

### Sample

The 2004 Pelotas (Brazil) Birth Cohort is a longitudinal, population-based, prospective study. It included all births from January 1st to December 31st from 2004 identified in the city’s maternity hospitals, comprising 4,231 newborns in the perinatal period ^27^ ^,^ ^28^. A total of nine follow-ups have been carried out so far: perinatal, at 3, 12, 24 and 48 months; and at 6, 11, 15 and 18 years of age. This study analyzed data from cohort participants who participated in follow-ups at ages 6 and 11, and a subset followed at 12 years old ^29^.

In 2017, a subsample of 1,303 participants from the 2004 cohort born between September and December was selected for follow-up. From this subsample, 497 participants were randomly selected within strata of body mass index (BMI) for age z-scores to include in the microbiome-referred substudy participants across the whole distribution of BMI. Exclusion criteria were severe cognitive impairment, due to potential difficulties in sample collection or answering the questionnaire, and a pregnancy or postpartum period of six months at the time of the interview. In total, 366 participants provided stool samples. More details on sample selection can be found in Supplementary Material (Figure S1; https://cadernos.ensp.fiocruz.br/static//arquivo/suppl-e00094424_9468.pdf) and are detailed elsewhere ^29^ ^,^ ^30^.

Questionnaires and stool samples were collected at the participant’s homes, in Pelotas. The adolescents, with the help of their mothers or guardians, collected stool samples after being briefed by the interviewers, using a Norgen Biotek plastic tube (https://norgenbiotek.com) and a collection kit as previously described ^29^ ^,^ ^30^.

### Fecal samples processing

Following collection, the samples were sent to the Centre for the Analysis of Genome Evolution & Function (CAGEF) at the University of Toronto (Canada) for molecular and bioinformatics analyses. Of the 366 samples collected, one could not be shipped to Canada due to participant ethical authorization issues, and another failed during amplification and sequencing, resulting in a total of 364 samples available for analysis.

DNA was isolated using the ZymoBIOMICS DNA Miniprep Kit (product n. D4300, Zymo Research; https://www.zymoresearch.com). The V3V4 region of the 16S rRNA gene was then tripled with barcoded primers 338F and 806R to enable multiplex sequencing ^31^ ^,^ ^32^. The products were normalized, purified with Ampure XP beads, and sequenced on the Illumina MiSeq platform (Illumina; https://www.illumina.com/) using V3 chemistry (2 x 300 bp).

For quality control, a single-species (*Pseudomonas aeruginosa* DNA), a mock community (Zymo Microbial Standard), and a negative control without a DNA template were included. Sequence data were processed using the UNOISE pipeline in USEARCH v11.0.667 and vsearch v2.10.4 ^33^ ^,^ ^34^ ^,^ ^35^. Sequences were trimmed at the 3’ ends based on a Q15 quality threshold using cutadapt v1.18. They were then filtered for quality using criteria such as a maximum expected error of 1.0, and length constraints of 100 to 600 base pairs. Afterward, sequences underwent de-replication, singleton removal, denoising, and chimera filtering using the unoise3 command. Operational taxonomic units (OTU) were assigned at 99% identity, and taxonomy was determined with the SINTAX algorithm and RDP database version 16, with a confidence threshold of 0.8 ^36^.

OTU sequences were aligned using QIIME1 (v1.9.1), a version that has since been updated. The comprehensive protocol is detailed in a previously published source ^29^.

### Gut microbiota analysis

Alpha diversity (Chao1, Simpson’s, and Shannon Entropy) and beta diversity metrics (weighted and unweighted UniFrac distances) were calculated using the QIIME2 software ^37^.

Taxa with more than 5% of zeros were analyzed for presence/absence associations in a binary model using hurdle analysis, where non-zero counts were transformed into 1. Taxa with at least 95% of observations were analyzed using their relative abundance in quantitative model and the data was transformed and normalized as presented elsewhere ^38^. The transformation consisted of calculating the proportions of zero for each taxon and then applying an inverse rank normal transformation to the data, using the *qnorm* function of the R program (https://www.r-project.org/).

To better understand the relationship between ultra-processed foods and the microbiota, we performed an association analysis at the phylum and genus levels. Relative abundance of phyla widely detected in the human gut (former *Actinobacteria*, *Bacteroidetes*, *Firmicutes*, and *Proteobacteria*) were evaluated. Those phyla had their nomenclature recently reviewed: *Actinomycetota*, *Bacteroidota*, *Bacillota* and *Pseudomonadota*, respectively ^39^. In this study, we chose to describe the data using the previous nomenclature to facilitate comparison with earlier published studies. Only genera present in more than 20% of the sample were included in the relative abundance analysis.

### Food consumption and ultra-processed food

This study collected dietary data from participants at ages 6, 11, and 12 using food frequency questionnaires (FFQ) with a 12-month recall period, which embeds a more holistic approach to assess diet quality, rather than focus on isolated nutrients. At age 6, the FFQ included 54 items, completed by the mother or guardian, and was validated based on three 24-hour dietary recalls. For the 11 and 12-year follow-ups, the FFQ expanded to 89 items ^40^. At 11, the respondent was the adolescent’s mother or guardian, and at 12 years old, it was the adolescent. The FFQ inquired about the frequency and portion size of each food item, with portion sizes based on standard Brazilian household measures and visually presented to participants. This approach enabled a detailed assessment of the participants’ dietary habits over these periods ^41^.

Participants indicated their consumption frequency for various food items ranging from “never or less than once a month” to “five or more times a day”. These frequencies were then converted into annual consumption rates and divided by 365.25 to estimate daily intake. The amount in grams (g) of each food was calculated according to the frequency of daily consumption and the reported portion size. Portions were adjusted by halving or increasing by 50% depending on whether they were smaller or larger than the standard portion, respectively. All foods were then categorized into one of four levels of processing according to the NOVA classification ^42^. The foods investigated in the FFQs are presented in Supplementary Material (Box S1; https://cadernos.ensp.fiocruz.br/static//arquivo/suppl-e00094424_9468.pdf).

Among the assessed foods, 18 were classified as ultra-processed foods at 6 years old, while at ages 11 and 12, this classification was extended to 26 items. The proportion in grams from each category was then calculated in relation to total food consumption. The use of grams is justified as it reflects absolute intake, correlating more directly with the body’s physical impact. To evaluate the effect of food consumption on microbiota, absolute quantity could be more pertinent, as gastrointestinal effects, such as carbohydrate fermentation and additive absorption, are more likely influenced by the total amount of food consumed than by the proportion of calories. For our analysis, ultra-processed food consumption, measured in grams, was divided into tertiles ^43^.

### Covariates

The covariates used for model adjustment were selected based on a theoretical framework, prioritizing variables with established evidence in the literature regarding their association with ultra-processed food consumption and gut microbiota. The covariates encompassed maternal and participants’ characteristics obtained in the perinatal assessment: maternal age (≤ 24 years, 25-34 years, ≥ 35 years), parity (1, 2-3, or ≥ 4 live births), total family income (in quintiles), child sex (female/male), gestational age (≤ 36 weeks, 37-41 weeks), type of delivery (vaginal/cesarean section), and birth weight (< 2,500g, 2,500-3,499g, > 3,500g).

Additional variables were breastfeeding duration (< 12 months, 12-24 months, > 24 months), as recorded at the 12-month follow-up; participant’s skin color (white, brown, black, or other), as reported by the mother at the 6-year follow-up; antibiotic use within six months previous to the interview, ascertained at the 12-year follow-up (yes/no); and BMI distributed in tertiles. Consumption of other food categories from the NOVA classification (minimally processed foods, processed culinary ingredients, and processed foods), was also incorporated into the analyses. The regression models were adjusted simultaneously for these variables.

### Statistical analyses

Linear regression models were employed to evaluate the relationship between tertiles of ultra-processed food consumption and alpha diversity indices (Chao1 Diversity Index, Simpson Diversity Index, and Shannon Entropy). The results were presented as β coefficients with a 95% confidence interval (95%CI). The beta diversity was measured using unweighted UniFrac and weighted UniFrac distance matrices from OTU tables generated in QIIME2 ^37^. The analysis of variance was evaluated by 999 permutations (PERMANOVA) tests, the effects were evaluated using adonis (in the *vegan* package, in RStudio; https://rstudio.com/) ^44^.

Regarding relative abundance outcomes, associations between ultra-processed food consumption and abundance at the phylum level were assessed using linear regression models. For taxa classified as present or absent according to the criteria outlined (binary model), logistic regression was employed. Conversely, for taxa where relative abundance was treated as a continuous variable (quantitative model), linear regression models were utilized to evaluate the associations.

All analyses were conducted separately for each time (ages 6, 11, and 12). Crude and adjusted models were employed to ascertain the associations between exposure (ultra-processed foods) and outcomes (gut microbiota composition). In all adjusted models, both maternal and adolescent covariables were included. A false discovery rate (FDR) of 0.05 was used to correct the p-values from the adjusted (main) models for each table and account for the multiple testing burden.

All analyses were conducted using Stata software, version 16.0 (https://www.stata.com), except for those involving beta diversity, as described above.

### Ethical aspects

The 2004 Pelotas (Brazil) Birth Cohort microbiome study was approved by Research Ethics Committee of the School of Medicine of the Federal University of Pelotas (registration n. 1,896,438) and by the Brazilian National Research Ethics Committee (registration n. 2,372,760). The participants’ mothers or guardians signed the informed consent form and the adolescent signed the assent form. The study was approved by the University of Toronto (protocol #: 00036176), and the Hospital for Sick Children Research Ethics Board (1000059180).

The study was registered at the Brazilian National System for Genetic Heritage and Associated Traditional Knowledge Management (protocol IDs A0C82E7 and R79C01C). Shipment of samples from the Federal University of Pelotas to the University of Toronto was conducted under a Material Transfer Agreement, in compliance with Brazilian ethical regulations.

## Results

### Participants

A total of 364 participants were assessed in the microbiome sub-study (Supplementary Material - Figure S1; https://cadernos.ensp.fiocruz.br/static//arquivo/suppl-e00094424_9468.pdf). All participants possessed data from the microbiome (12 years), as well as from the 6- and 11-year follow-ups, rendering them eligible for inclusion in this investigation. Table 1 describes the characteristics of the included sample.

Table 1 Description of the sample according to household, maternal, and cohort participants characteristics from the perinatal period to age 12. The 2004 Pelotas (Brazil) Birth Cohort (n = 364).

**Table:** 

Covariables	n (%)
Perinatal and maternal characteristics	
Mother’s age (years) (n = 364)	
≤ 24	174 (47.8)
25-34	148 (40.6)
≥ 35	42 (11.5)
Gestational age (weeks) (n = 362)	
< 36	26 (7.2)
37-41	336 (92.8)
Type of delivery (n = 364)	
Vaginal	185 (50.8)
Cesarean	179 (49.2)
Birth weight (g) (n = 364)	
< 2,500	31 (8.5)
2,500-3,499	225 (61.8)
> 3,500	108 (29.7)
Duration of breastfeeding (months) (n = 362)	
< 12	225 (62.2)
12-24	64 (17.7)
> 24	73 (20.2)
Parity (n = 363)	
1	157 (43.3)
2-3	151 (41.6)
4 or more	55 (15.2)
Household income (quintiles) (n = 364)	
1st (lower)	80 (22.0)
2nd	71 (19.5)
3rd	68 (18.7)
4th	77 (21.2)
5th	68 (18.7)
Participant’s characteristics	
Sex (n = 364)	
Male	192 (52.8)
Female	172 (47.3)
Skin color (n = 363)	
White	247 (68.0)
Black	45 (12.4)
Brown	64 (17.6)
Others	7 (1.9)
Use of antibiotics in the last six months at age 12 (n = 363)	
No	285 (78.5)
Yes	78 (21.5)

During the perinatal period, almost half of mothers were younger than 24 years old (47.8%) and 56.8% reported having two or more previous deliveries (Table 1). More than half of participants are male (52.5%) and self-reported white skin color (68%). Most participants were born between the 37th and 41st weeks of gestation (92.8%), by vaginal delivery (50.8%), weighing between 2,500 and 3,499g (61.8%), and were breastfed for less than 12 months (62.2%) (Table 1). The mean BMI was 17.10kg/m^2^ (standard deviation - SD = 3.40) at 6 years old, 19.97kg/m^2^ (SD = 5.25) at 11 years old and 21.49kg/m^2^ (SD = 5.41) at 12 years old (data not shown).

### Ultra-processed food consumption characterization

Table 2 presents the median (interquartile range - IQR) of daily food consumption in grams according to the level of food processing. At the age of 6, the median daily ultra-processed food consumption was 1,023.01g (IQR: 655.13; 1,664.10), at 11 it was 644.67g (IQR: 418.72; 1,001.94), and at 12, it decreased to 541.87g (IQR: 327.76; 852.63).

**Table 2:** Median of daily food intake (in grams) consumed by cohort participants at 6, 11 and 12 years of age, according to the level of food processing (NOVA classification). The 2004 Pelotas (Brazil) Birth Cohort.

NOVA classification	6 years old (n = 354)	11 years old (n = 364)	12 years old (n = 364)
Ultra processed food			
1st tertile			
Median (g)	540.74	312.24	234.37
Interquartile range (g)	421.11; 618.86	226.35; 419.96	154.50; 328.37
2nd tertile			
Median (g)	920.20	644.76	542.98
Interquartile range (g)	795.43; 1,053.12	583.03; 750.33	454.86; 620.98
3rd tertile			
Median (g)	1,582.62	1,289.42	1,036.50
Interquartile range (g)	1,376.18; 2,113.78	1,007.68; 1,764.45	859.17; 1,449.71
In natura			
Median (g)	1,476.96	1,732.59	1,357.72
Interquartile range (g)	1,121.87; 1,989.43	1,206.89; 2,298.84	1,004.43; 1,927.74
Processed culinary ingredients			
Median (g)	10.95	16.18	7.5
Interquartile range (g)	0.00; 51.26	4.27; 32.70	0.49; 23.84
Processed food			
Median (g)	103.28	134.41	91.00
Interquartile range (g)	52.53; 164.52	96.60; 170.50	59.37; 136.45

### Gut microbiota diversity and composition characterization

The relative abundance of taxa observed in the samples has been previously documented ^29^. The most prevalent genus was *Bifidobacterium*, detected in 94.8% of participants, while the least common genus was *Megasphaera*, observed in 20% of participants (Table 3). Table 4 illustrates the average alpha diversity indices stratified by the covariates, the mean and standard deviation of the indexes, respectively, are Chao1 = 367.107 (SD = 99.75); Simpson Eveness: mean = 0.14 (SD = 0.05); Shannon Entropy: mean = 1.82 (SD = 0.10). Table 5 illustrates the relative abundance of phyla stratified by the covariates.

**Table 3:** Description of the taxa included and the model to which they belong.

Phylum	Genus	Model	Mean (SD)	n (%)
*Actinobacteria*	-	Q	0 (0.996)	-
*Bacteroidetes*	-	Q	0 (0.996)	-
*Firmicutes*	-	Q	0 (0.996)	-
*Proteobacteria*	-	Q	0 (0.996)	-
-	*Bacteroides*	Q	0 (0.996)	-
-	*Parabacteroides*	Q	0 (0.991)	-
-	*Prevotella*	Q	0 (0.992)	-
-	*Alistipes*	Q	0 (0.989)	-
-	*Clostridium_Sensu_Stricto*	Q	0 (0.996)	-
-	*Anaerostipes*	Q	0 (0.996)	-
-	*Blautia*	Q	0 (0.996)	-
-	*Clostridium_xlva*	Q	0 (0.996)	-
-	*Coprococcus*	Q	0 (0.992)	-
-	*Dorea*	Q	0 (0.996)	-
-	*Fusicatenibacter*	Q	0 (0.994)	-
-	*Roseburia*	Q	-	-
-	*Ruminococcus2*	Q	0 (0.996)	-
-	*Intestinibacter*	Q	0 (0.988)	-
-	*Romboutsia*	Q	0 (0.993)	-
-	*Clostridium_IV*	Q	0 (0.996)	-
-	*Faecalibacterium*	Q	0 (0.996)	-
-	*Gemmiger*	Q	0 (0.989)	-
-	*Oscillibacter*	Q	0 (0.995)	-
-	*Lachnospiracea_incertae_sedis*	Q	0 (0.994)	-
-	*Ruminococcus*	Q	0 (0.993)	-
-	*Erysipelotrichaceae_incertae_sedis*	B	-	140 (38.5)
-	*Methanobrevibacter*	B	-	229 (62.9)
-	*Methanosphaera*	B	-	75 (20.6)
-	*Actinomyces*	B	-	176 (48.4)
-	*Rothia*	B	-	75 (20.6)
-	*Bifidobacterium*	B	-	345 (94.8)
-	*Collinsella*	B	-	344 (94.5)
-	*Eggerthella*	B	-	164 (45.1)
-	*Gordonibacter*	B	-	95 (43.4)
-	*Olsenella*	B	-	158 (43.4)
-	*Senegalimassilia*	B	-	197 (54.1)
-	*Slackia*	B	-	238 (65.4)
-	*Butyricimonas*	B	-	279 (76.7)
-	*Odoribacter*	B	-	335 (21.4)
-	*Porphyromonas*	B	-	78 (21.4)
-	*Paraprevotella*	B	-	162 (44.5)
-	*Lactobacillus*	B	-	222 (61.0)
-	*Streptococcus*	B	-	343 (94.2)
-	*Christensenella*	B	-	78 (21.4)
-	*Mogibacterium*	B	-	91 (25.0)
-	*Eubacterium*	B	-	306 (84.1)
-	*Butyrivibrio*	B	-	133 (36.5)
-	*Clostridium_xlvb*	B	-	334 (91.8)
-	*Eisenbergiella*	B	-	177 (48.6)
-	*Howardella*	B	-	129 (35.4)
-	*Peptococcus*	B	-	94 (25.8)
-	*Peptoniphilus*	B	-	87 (24.0)
-	*Peptostreptococcus*	B	-	85 (23.4)
-	*Terrisporobacter*	B	-	91 (25.0)
-	*Anaerofilum*	B	-	171 (47.0)
-	*Anaerotruncus*	B	-	303 (83.2)
-	*Flavonifractor*	B	-	289 (79.4)
-	*Intestinimonas*	B	-	136 (37.4)
-	*Pseudoflavonifractor*	B	-	77 (21.2)
-	*Catenibacterium*	B	-	177 (48.6)
-	*Clostridium_XVIII*	B	-	304 (83.5)
-	*Holdemanella*	B	-	247 (67.9)
-	*Holdemania*	B	-	169 (46.4)
-	*Turicibacter*	B	-	315 (86.5)
-	*Phascolarctobacterium*	B	-	280 (76.9)
-	*Allisonella*	B	-	150 (41.2)
-	*Dialister*	B	-	230 (63.2)
-	*Megamonas*	B	-	91 (25.0)
-	*Megasphaera*	B	-	71 (20.0)
-	*Mitsuokella*	B	-	83 (22.8)
-	*Veillonella*	B	-	259 (71.2)
-	*Victivallis*	B	-	235 (64.6)
-	*Parasutterella*	B	-	257 (70.6)
-	*Sutterella*	B	-	232 (63.7)
-	*Bilophila*	B	-	315 (76.9)
-	*Desulfovibrio*	B	-	280 (76.9)
-	*Campylobacter*	B	-	90 (24.7)
-	*Haemophilus*	B	-	218 (59.9)
-	*Akkermansia*	B	-	235 (64.6)

B: binary model; Q: quantitative model; SD: standard deviation.

**Table 4:** Average alpha diversity indices, according to covariates in a subsample of the 2024 Pelotas (Brazil) Birth Cohort.

Covariables	Chao1		Simpson eveness		Shannon entropy	
	Average (SD)	p-value	Average (SD)	p-value	Average (SD)	p-value
Perinatal and maternal characteristics						
Sex (n = 364)		0.231		0.326		0.618
Male	373.0 (7.05)		0.1 (0.05)		1.8 (0.1)	
Female	360.5 (7.77)		0.1 (0.04)		1.8 (0.1)	
Skin color (n = 363)		0.0001		0.196		0.006
White	352.8 (93.75)		0.1 (0.05)		1.8 (0.1)	
Black	418.3 (100.01)		0.1 (0.06)		1,8 (0.1)	
Brown	381.4 (107.51)		0.1 (0.04)		1.8 (0.1)	
Others	433.0 (90.87)		0.2 (0.05)		1.9 (0.1)	
Mother’s age (years) (n = 364)		0.008		0.874		0.051
≤ 24	380.3 (94.77)		0.1 (0.05)		1.8 (0.1)	
25-34	347.7 (99.88)		0.1 (0.05)		1,8 (0.1)	
≥ 35	380.6 (110.20)		0.1 (0.05)		1.8 (0.1)	
Gestational age (weeks) (n = 362)		0.118		0.533		0.070
≤ 36	396.8 (101.49)		0.1 (0.03)		1.9 (0.1)	
37-41	365.0 (99.33)		0.1 (0.04)		1.8 (0.1)	
Type of delivery (n = 364)		0.0005		0.714		0.006
Vaginal	384.8 (104.57)		0.1 (0.05)		1.8 (0.1)	
Cesarean	348.8 (91.25)		0.1 (0.05)		1.8 (0.1)	
Birth weight (g) (n = 364)		0.964		0.726		0.892
< 2,500	364.8 (113.18)		0.1 (0.04)		1.8 (0.1)	
2,500-3,499	366.4 (100.65)		0.1 (0.05)		1.8 (0.1)	
> 3,500	369.2 (94.59)		0.1 (0.05)		1.8 (0.1)	
Duration of breastfeeding (months) (n = 362)		0.100		0.943		0.144
< 12	368.6 (93.71)		0.1 (0.05)		1.8 (0.1)	
12-24	384.7 (106.75)		0.4 (0.04)		1.8 (0.1)	
> 24	349.0 (109.43)		0.1 (0.05)		1.8 (0.1)	
Parity (n = 363)		0.005		0.109		0.466
1	361.0 (93.58)		0.1 (0.05)		1.8 (0.1)	
2-3	358.1 (99.71)		0.1 (0.05)		1.8 (0.1)	
4 or more	407.0 (108.14)		0.1 (0.05)		1.8 (0.1)	
Household income (quintiles) (n = 364)		0.001		0.572		0.087
1st (lower)	389.4 (106.97)		0.1 (0.04)		1.8 (0.1)	
2nd	384.8 (110.11)		0.1 (0.05)		1.8 (0.1)	
3rd	376.1 (99.05)		0.1 (0.04)		1.8 (0.1)	
4th	353.5 (86.37)		0.1 (0.05)		1.8 (0.1)	
5th	328.8 (82.27)		0.1 (0.05)		1.8 (0.1)	
Adolescent characteristics						
Follow-up at age 6						
Grams of in natura (n = 354)		0.675		0.889		0.301
Less than or equal to median	364.8 (7.87)		0.1 (0.05)		1.8 (0.1)	
Greater than median	369.3 (7.16)		0.1 (0.05)		1.8 (0.1)	
Grams of ingredients (n = 354)		0.003		0.701		0.021
Less than or equal to median	351.7 (96.52)		0.1 (0.05)		1.8 (0.1)	
Greater than median	382.8 (7.68)		0.1 (0.05)		1.8 (0.1)	
Grams of processed food (n = 354)		0.044		0.309		0.103
Less than or equal to median	356.3 (93.10)		0.1 (0.05)		1.8 (0.1)	
Greater than median	377.7 (105.52)		0.1 (0.05)		1.8 (0.1)	
BMI (tertiles) (n = 339)		0.0005		0.824		0.007
1st (lower)	387.0 (104.87)		0.1 (0.05)		1.8 (0.1)	
2nd	376.3 (97.42)		0.1 (0.04)		1.8 (0.1)	
3rd	337.8 (94.46)		0.1 (0.05)		1.7 (0.1)	
Follow-up at age 11						
Grams of in natura (n = 364)		0.018		0.252		0.281
Less than or equal to median	354.7 (98.71)		0.1 (0.05)		1.8 (0.1)	
Greater than median	379.4 (99.36)		0.1 (0.05)		1.8 (0.1)	
Grams of ingredients (n = 364)		0.016		0.796		0.091
Less than or equal to median	354.5 (95.18)		0.1 (0.05)		1.8 (0.1)	
Greater than median	379.6 (102.82)		0.1 (0.05)		1.8 (0.1)	
Grams of processed food (n = 364)		0.022		0.608		0.019
Less than or equal to median	355.2 (340.43)		0.1 (0.05)		1.8 (0.1)	
Greater than median	379.2(97.12)		0.1 (0.05)		1.8 (0.1)	
BMI (tertiles) (n = 363)		< 0.001		0.614		0.001
1st (lower)	389.2 (103.63)		0.1 (0.05)		1.8 (0.1)	
2nd	379.6 (96.98)		0.1 (0.04)		1.8 (0.1)	
3rd	331.9 (89.13)		0.1 (0.05)		1.7 (0.1)	
Follow-up at age 12						
Grams of in natura (n = 364)		0.089		0.409		0.495
Less than or equal to median	348.7 (91.79)		0.1 (0,05)		1.8 (0.1)	
Greater than median	371.4 (101.19)		0.1 (0.05)		1.8 (0.1)	
Grams of ingredients (n = 364)		0.088		0.643		0.353
Less than or equal to median	358.2 (99.18)		0.1 (0.05)		1.8 (0.1)	
Greater than median	376.1 (99.79)		0.1 (0.05)		1.8 (0.1)	
Grams of processed food (n = 364)		0.007		0.191		0.177
Less than or equal to median	352.9 (94.30)		0.1 (0.13)		1.8 (0.1)	
Greater than median	381.2 (103.21)		0.1 (0.13)		1.8 (0.1)	
BMI (tertiles) (n = 357)		0.0001		0.585		0.005
1st (lower)	389.6 (104.84)		0.1 (0.05)		1.8 (0.1)	
2nd	376.3 (91.82)		0.1 (0.04)		1.8 (0.1)	
3rd	336.8 (95.94)		0.1 (0.05)		1.7 (0.1)	
Use of antibiotics in the last six months at age 12 (n = 363)		0.019		0.958		0.086
No	373.4 (99.34)		0.1 (0.05)		1.8 (0.1)	
Yes	343.5 (98.93)		0.1 (0.05)		1.8 (0.1)	

BMI: body mass index; SD: standard deviation.

**Table 5:** Relative abundance of gut microbiome, according to covariates in a subsample of the 2004 Pelotas (Brazil) Birth Cohort.

Covariables	*Actinobacteria*		*Bacteroidetes*		*Firmicutes*		*Proteobacteria*	
	Mean (SD)	p-value	Mean (SD)	p-value	Mean (SD)	p-value	Mean (SD)	p-value
Perinatal and maternal characteristics								
Sex (n = 364)		0.810		0.432		0.803		0.156
Male	-0.012 (0.07)		0.039 (1.03)		-0.012 (1.06)		-0.070 (0.96)	
Female	0.013 (0.08)		-0.043 (0.96)		0.014 (0.92)		0.078 (1.03)	
Skin color (n = 363)		0.242		0.604		0.191		0.131
White	-0.065 (1.01)		-0.037 (0.98)		0.066 (0.98)		-0.030 (0.96)	
Black	0.240 (1.05)		-0.004 (0.97)		-0.077 (0.91)		0.224 (1.02)	
Brown	0.082 (0.91)		0.150 (1.12)		-0.216 (1.13)		0.047 (1.08)	
Others	-0.120 (0.72)		-0.111 (0.66)		0.241 (0.63)		-0.650 (1.18)	
Mother’s age (years) (n = 364)		0.081		0.439		0.847		0.111
≤ 24	0.029 (0.95)		0.046 (0.95)		-0.057 (0.91)		0.005 (1.09)	
25-34	-0.063 (0.98)		0.016 (1.03)		0.017 (1.04)		-0.052 90.97)	
≥ 35	0.307 (1.12)		-0.182 (0.88)		0.009 (0.92)		0.296 (0.94)	
Gestational age (weeks) (n = 362)		0.044		0.437		0.195		0.559
≤ 36	0.385 (0.16)		0.144 (0.92)		-0.242 (0.80)		0.108 (1.11)	
37-41	-0.021 (0.05)		-0.014 (1.00)		0.021 (1.01)		-0.011 (0.99)	
Type of delivery (n = 364)		0.764		0.234		0.149		0.425
Vaginal	0.015 (0.91)		0.061 (0.97)		-0.074 (0.94)		0.041 (1.02)	
Cesarean	-0.016 (0.08)		-0.063 (1.02)		0.077 (1.04)		-0.042 (0.98)	
Birth weight (g) (n = 364)		0.694		0.519		0.313		0.385
< 2,500	-0.054 (1.21)		0.182 (0.98)		-0.257 (0.98)		0.087 (1.03)	
2,500-3,499	0.035 (0.95)		-0.0006 (1.01)		0.014 (1.02)		-0.057 (0.99)	
> 3,500	-0.058 (1.02)		-0.0511 (0.97)		0.045 (0.94)		0.093 (0.99)	
Duration of breastfeeding (months) (n = 362)		0.068		0.574		0.896		0.030
< 12	0.084 (0.99)		-0.020 (1.01)		0.010 (1.00)		-0.069 (0.94)	
12-24	-0.234 (0.96)		-0.061 (0.98)		0.023 (1.01)		0.301 (1.17)	
> 24	-0.061 (1.02)		0.103 (0.98)		-0.046 (1.00)		-0.031 (0.96)	
Parity (n = 363)		0.236		0.394		0.047		0.707
1	-0.046 (1.02)		-0.070 (0.98)		0.105 (0.98)		0.014 (0.99)	
2-3	-0.038 (0.94)		0.033 (1.00)		-0.006 (0.99)		-0.044 (0.96)	
4 or more	0.204 (1.06)		0.129 (1.04)		-0281 (1.03)		0.082 (1.12)	
Household income (quintiles) (n = 364)		0.007		0.116		0.007		0.803
1st (lower)	0.284 (0.92)		0.222 (0.97)		-0.337 (0.90)		0.053 (1.02)	
2nd	0.046 (0.96)		0.069 (1.05)		-0.097 (1.07)		0.027 (0.96)	
3rd	0.129 (0.93)		-0.184 (0.99)		0.143 (0.95)		-0.096 (1.19)	
4th	-0.268 (0.93)		0.0009 (1.00)		0.095 (1.02)		-0.067(0.90)	
5th	-0.117 (1.17)		-0.151 (0.92)		0.215 (0.93)		0.081 (0.96)	
Adolescent characteristics								
Follow-up at age 6								
Grams of in natura (n = 354)		0.927		0.452		0.350		0.890
Less than or equal to median	-0.002 (1.04)		0.027 (1.01)		-0.038 (0.98)		0.0007 (0.94)	
Greater than median	-0.012 (0.97)		-0.052 (0.99)		0.061 (1.00)		0.015 (1.05)	
Grams of ingredients (n = 354)		0.966		0.236		0.459		0.164
Less than or equal to median	-0.005 (1.02)		0.049 (1.04)		0.027 (1.01)		-0.065 (1.03)	
Greater than median	-0.009 (0.08)		-0.076 (0.95)		0.051 (0.97)		0.083 (0.95)	
Grams of processed food (n = 354)		0.163		0.029		0.095		0.891
Less than or equal to median	-0.080 (0.07)		0.103 (0.99)		-0.076 (1.01)		0.0009 (0.99)	
Greater than median	0.067 (1.02)		-0.128 (0.99)		0.099 (0.96)		0.015 (1.00)	
BMI (tertiles) (n = 339)		0.333		0.458		0.735		0.376
1st (lower)	0.064 (1.01)		-0.071 (0.94)		0.050 (0.948)		-0.018 (1.03)	
2nd	0.009 (0.96)		-0.033 (1.01)		0.014 (0.96)		-0.058 (0.94)	
3rd	-0.098 (1.00)		0.087 (1.02)		-0.051 (1.06)		0.120 (1.02)	
Follow-up at age 11								
Grams of in natura (n = 364)		0.416		0.218		0.102		0.293
Less than or equal to median	-0.043 (1.02)		-0.065 (0.91)		0.086 (0.96)		0.055 (0.89)	
Greater than median	0.042 (0.07)		0.064 (1.02)		-0.085 (1.02)		-0.055 (1.09)	
Grams of ingredients (n = 364)		0.083		0.071		0.030		0.818
Less than or equal to median	-0.091 (1.04)		0.095 (1.06)		0.114 (1.03)		0.012 (0.94)	
Greater than median	0.090 (0.07)		0.094 (0.92)		-0.112 (0.95)		-0.012 (1.05)	
Grams of processed food (n = 364)		0.024		0.162		0.040		0.416
Less than or equal to median	-0.117 (1.05)		-0.073 (0.93)		0.106 (0.07)		-0.042 (0.99)	
Greater than median	0.118 (0.92)		0.074 (1.06)		-0.107 (1.01)		0.043 (1.00)	
BMI (tertiles) (n = 363)		0.097		0.297		0.409		0.615
1st (lower)	0.088 (0.98)		-0.029 (0.90)		-0.016 (0.90)		0.018 (1.07)	
2nd	0.070 (0.97)		-0.082 (0.98)		0.093 (0.98)		-0.071 (0.85)	
3rd	-0.159 (1.02)		0.111 (1.09)		-0.076 (1.09)		0.052 (1.06)	
Follow-up at age 12								
Grams of in natura (n = 364)		0.691		0.746		0.757		0.006
Less than or equal to median	-0.043 (0.94)		0.035 (0.95)		-0.033 (0.93)		0.294 (0.97)	
Greater than median	0.010 (1.01)		-0.008 (1.01)		0.008 (1.01)		-0.069 (0.99)	
Grams of ingredients (n = 364)		0.061		0.094		0.022		0.427
Less than or equal to median	-0.097 (1.01)		-0.087 (0.97)		0.118 (0.97)		0.041 (0.97)	
Greater than median	0.098 (0.98)		0.088 (1.01)		-0.119 (1.01)		-0.042 (1.02)	
Grams of processed food (n = 364)		0.118		0.948		0.308		0.363
Less than or equal to median	-0.082 (1.02)		-0.003 (0.94)		0.054 (0.93)		0.048 (0.98)	
Greater than median	0.081 (0.97)		0.003 (1.05)		-0.053 (1.06)		-0.047 (1.02)	
BMI (tertiles) (n = 357)		0.142		0.319		0.544		0.699
1st (lower)	0.052 (1.01)		-0.015 (0.95)		-0.016 (0.95)		-0.021 (1.03)	
2nd	0.102 (0.92)		-0.082 (1.03)		0.074 (1.00)		-0.059 (0.94)	
3rd	-0.139 (1.05)		0.109 (1.00)		-0.067 (1.04)		0.048 (1.01)	
Use of antibiotics in the last six months at age 12 (n = 363)		0.070		0.881		0.381		0.791
No	0.047 (0.98)		0.008 (1.01)		-0.028 (1.00)		0.009 (1.01)	
Yes	-0.184 (0.12)		-0.010 (0.93)		0.083 (0.98)		-0.024 (0.95)	

BMI: body mass index; SD: standard deviation.

### Ultra-processed food consumption and gut microbiota composition

Table 6 presents the associations between ultra-processed food consumption and alpha diversity indices. In the crude analysis, a significant association was observed between ultra-processed food consumption and the Chao1 index for the highest tertile at age 11 (β = 29.819; 95%CI: 4.87; 54.77) and for both tertiles at age 12 (p = 0.016). Shannon entropy was also associated with highest tertile of consumption at age 11 (β = 0.028; 95%CI: 0.001; 0.050). After adjusting for covariates, only the association with the third tertile at age 11 persisted for both metrics. No significant associations between ultra-processed food consumption and alpha diversity were found at ages 6, 11, and 12 after multiple testing correction.

**Table 6:** Crude and adjusted linear regression showing the association of ultra-processed foods consumption at ages 6, 11, and 12 years with gut microbiota composition (alpha diversity indices). The 2004 Pelotas (Brazil) Birth Cohort.

Exposure: ultra-processed foods consumption	Crude model		Adjusted model *		FDR p-value **
	β (95%CI)	p-value **	β (95%CI)	p-value **	
Outcome: Chao1					
6 years		0.339		0.551	0.620
1st tertile	Reference		Reference		
2nd tertile	5.898 (-19.70; 31.49)		-8.528 (-33.65; 16.59)		
3rd tertile	18.745 (-6.84; 44.34)		5.284 (-19.77; 30.34)		
11 years		0.016		0.039	0.176
1st tertile	Reference		Reference		
2nd tertile	-3.507 (-28.46; 21.45)		-10.584 (-35.57; 14.4)		
3rd tertile	29.819 (4.87; 54.77)		21.072 (-4.46; 46.61)		
12 years		0.016		0.257	0.463
1st tertile	Reference		Reference		
2nd tertile	25.899 (0.95; 50.85)		18.201 (-6.82; 43.22)		
3rd tertile	35.397 (10.45; 60.35)		18.411 (-6.83; 43.65)		
Outcome: Shannon eveness					
6 years		0.523		0.459	0.590
1st tertile	Reference		Reference		
2nd tertile	0.010 (-0.02; 0.04)		0.010 (-0.02; 0.04)		
3rd tertile	0.015 (-0.01; 0.04)		0.018 (-0.01; 0.05)		
11 years		0.020		0.037	0.176
1st tertile	Reference		Reference		
2nd tertile	-0.008 (-0.03; 0.02)		-0.014 (-0.04; 0.01)		
3rd tertile	0.028 (0.001; 0.05)		0.022 (-0.01; 0.05)		
12 years		0.096		0.459	0.590
1st tertile	Reference		Reference		
2nd tertile	0.018 (-0.01; 0.04)		0.010 (-0.02; 0.04)		
3rd tertile	0.029 (0.02; 0.06)		0.018 (-0.01; 0.05)		
Outcome: Simpson entropy					
6 years		0.945		0.177	0.398
1st tertile	Reference		Reference		
2nd tertile	0.007 (-0.09; 0.11)		0.006 (-0.10; 0.11)		
3rd tertile	-0.010 (-0.11; 0.09)		-0.010 (-0.11; 0.09)		
11 years		0.248		0.177	0.398
1st tertile	Reference		Reference		
2nd tertile	-0.070 (-0.17; 0.03)		-0.084 (-0.19; 0.02)		
3rd tertile	0.003 (-0.09; 0.10)		0.001 (-0.11; 0.11)		
12 years		0.783		0.846	0.846
1st tertile	Reference		Reference		
2nd tertile	0.033 (-0.07; 0.13)		0.029 (-0.07; 0.13)		
3rd tertile	0.007 (0.09; 0.11)		0.023 (-0.08; 0.13)		

95%CI: 95% confidence interval; FDR: false discovery rate.

Note: n = 334. Values in bold indicate p-value < 0.05.

* Models adjusted for perinatal variables (gestational age, type of delivery, birth weight, parity, total family income, sex, skin color), breastfeeding duration, other sources of consumption in the NOVA classification, body mass index and use of antibiotics six months before the interview at age 12;

** p-value for the test Parm.

**Figure 1:**
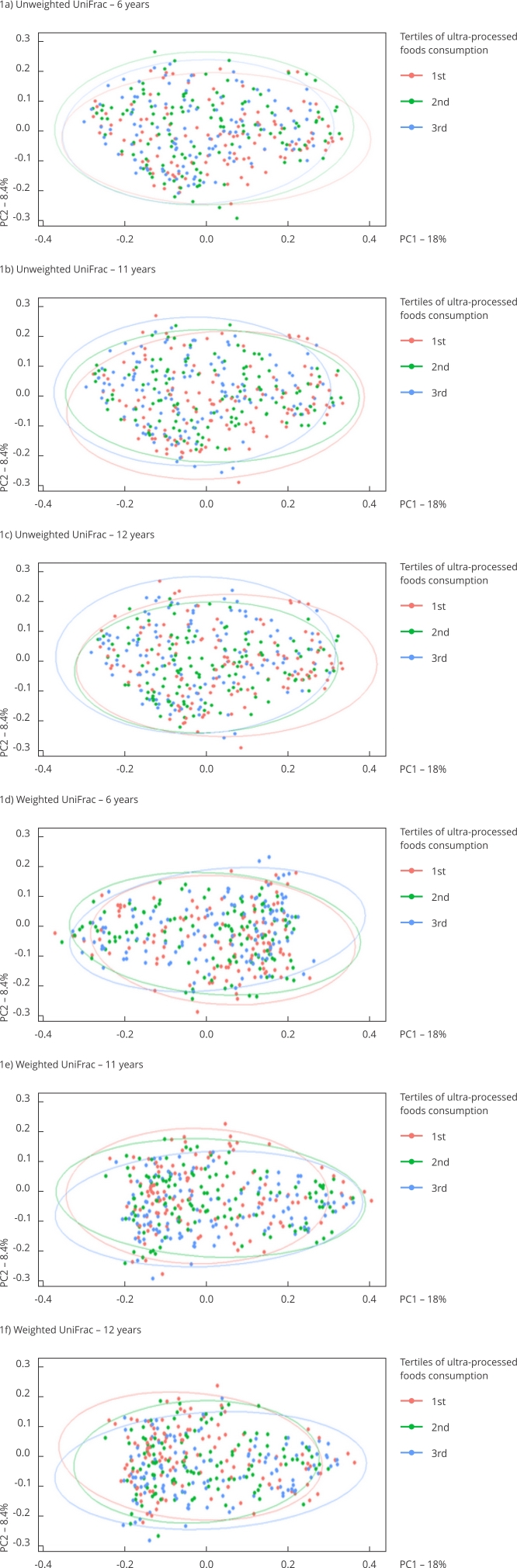
Principal coordinates analysis (PCoA) based on weighted and unweighted UniFrac distances for different levels of ultra-processed foods consumption in a sample aged 6, 11 and 12 years.

PC1: first principal component; PC2: second principal component.

Table 7 and Figure 1 display the results of the PERMANOVA analysis for beta diversity metrics. A significant association was observed between ultra-processed food consumption and the unweighted UniFrac metric at ages 11 (p = 0.007) and 12 (p = 0.001), accounting for 0.9% and 1.1% of the variation, respectively. No significant association was found for the weighted UniFrac metric.

**Table 7:** Comparison of gut microbiota beta diversity with ultra-processed foods consumption variables using the PERMANOVA test.

Variable	DF	Unweighted UniFrac		Weighted UniFrac	
		R^2^	p-value	R^2^	p-value
Ultra-processed foods consumption at 6 years	2	0.004	0.598	0.007	0.119
Ultra-processed foods consumption at 11 years	2	0.009	0.007	0.008	0.164
Ultra-processed foods consumption at 12 years	2	0.011	0.001	0.011	0.069

DF: degree of freedom.

Note: significance was set at p < 0.05.

Table 8 presents crude and adjusted analyses of relative abundance at the phylum level. Ultra-processed food consumption tertiles at 11 years of age were nominally associated with the phyla *Actinobacteria* and *Proteobacteria*. The former (p = 0.032) exhibited higher abundance in the highest ultra-processed food consumption tertile, while the latter (p = 0.045) showed an abundance decrease among ultra-processed food consumers.

**Table 8:** Results of the association between ultra-processed foods consumption and relative abundance at the phylum level using linear regressions at ages 6, 11, and 12 years. The 2004 Pelotas (Brazil) Birth Cohort.

Phylum	6 years	11 years	12 years
*Actinobacteria*			
Crude model			
Ultra-processed foods consumption (p-value *)	0.205	0.006	0.025
1st tertile	Reference	Reference	Reference
2nd tertile	0.076 (-0.18; 0.33)	0.041 (-0.21; 0.29)	0.703 (-0.18; 0.32)
3rd tertile	0.228 (-0.03; 0.07)	0.369 (0.12; 0.62)	0.330 (0.08; 0.58)
Adjusted model **			
Ultra-processed foods consumption (p-value */FDR)	0.499/0.626	0.032/0.090	0.19/0.784
1st tertile	Reference	Reference	Reference
2nd tertile	0.027 (-0.23; 0.29)	0.049 (-0.21; 0.31)	0.049 (-0.21; 0.31)
3rd tertile	0.146 (-0.11; 0.41)	0.325 (0.60; 0.59)	0.228 (-0.03; 0.49)
*Bacteroidetes*			
Crude model			
Ultra-processed foods consumption (p-value *)	0.553	0.900	0.555
1st tertile	Reference	Reference	Reference
2nd tertile	0.007 (0.25; 0.26)	0.005 (-0.25; 0.26)	0.068 (-0.18; 0.32)
3rd tertile	-0.122 (-0.38; 0.13)	-0.048 (-0.30; 0.20)	0.139 (-0.11; 0.39)
Adjusted model **			
Ultra-processed foods consumption (p-value */FDR)	0.499/0.626	0.436/0.550	0.941/0.941
1st tertile	Reference	Reference	Reference
2nd tertile	0.027 (-0.23; 0.29)	-0.078 (-0.45; 0.09)	0.046 (-0.22; 0.31)
3rd tertile	0.146 (-0.11; 0.41)	-0.178 (-0.45; 0.09)	0.034 (-0.23; 0.30)
*Firmicutes*			
Crude model			
Ultra-processed foods consumption (p-value *)	0.746	0.926	0.145
1st tertile	Reference	Reference	Reference
2nd tertile	-0.077 (-0.33; 0.18)	0.024 (-0.23; 0.28)	-0.066 (-0.32; 0.18)
3rd tertile	0.016 (-0.24; 0.27)	-0.027 (-0.28; 0.23)	-0.243 (-0.49; 0.01)
Adjusted model **			
Ultra-processed foods consumption (p-value */FDR)	0.626/0.626	0.550/0.550	0.792/0.941
1st tertile	Reference	Reference	Reference
2nd tertile	-0.088 (-0.35; 0.18)	0.126 (-0.14; 0.39)	-0.036 (-0.30; 0.23)
3rd tertile	0.040 (-0.22; 0.30)	0.135 (-0.14; 0.41)	-0.092 (-0.36; 0.17)
*Proteobacteria*			
Crude model			
Ultra-processed foods consumption (p-value *)	0.245	0.052	0.376
1st tertile	Reference	Reference	Reference
2nd tertile	0.024 (-0.23; 0.28)	-0.308 (-0.56; -0.06)	-0.065 (-0.32; 0.19)
3rd tertile	-0.175 (-0.43; 0.08)	-0.121 (-0.37; 0.13)	-0.177 (-0.43; 0.07)
Adjusted model **			
Ultra-processed foods consumption (p-value */FDR)	0.208/0.626	0.045/0.090	0.414/0.828
1st tertile	Reference	Reference	Reference
2nd tertile	0.014 (-0.29; 0.26)	-0.344 (-0.62; -0.07)	-0.006 (-0.28; 0.27)
3rd tertile	-0.220 (-0.49; 0.05)	-0.139 (-0.42; 0.14)	-0.164 (-0.44; 0.11)

FDR: false discovery rate.

* p-value for the test Parm;

** Model adjusted for perinatal variables (gestational age, type of delivery, birth weight, parity, total family income, sex, skin color), breastfeeding duration, other sources of consumption in the NOVA classification, body mass index and use of antibiotics six months before the interview at age 12.

Table 9 reports nominal associations between ultra-processed food consumption and relative abundances of genera for adjusted results. Supplementary Material - Table S1 (https://cadernos.ensp.fiocruz.br/static//arquivo/suppl-e00094424_9468.pdf) provides a comprehensive list of included genera, along with results from the crude and adjusted regression models. The genera with associations in more than one assessment were *Bacteroides* and *Peptostreptococcus*. Ultra-processed food consumption was inversely associated with the relative abundance of *Bacteroides* at 6 and 11 years of age. In contrast, *Peptostreptococcus* exhibited a nominal association with ultra-processed food consumption in both occasions, although the direction of the relationship was not consistent. However, none of these associations remained statistically significant after adjusting for multiple testing.

**Table 9:** Nominal results of the associations between ultra-processed foods consumption and relative abundance at the genus level at ages 6, 11, and 12 years. The 2004 Pelotas (Brazil) Birth Cohort.

Taxa (model)	Adjusted model *		FDR p-value
	β or OR (95%CI)	p-value ** (< 0.05)	
Exposure: ultra-processed foods consumption at 6 years			
*Bacteroides* *** (Q)		0.037	0.666
1st tertile	Reference		
2nd tertile	-0.133 (-0.37; 0.11)		
3rd tertile	-0.315 (-0.56; -0.07)		
*Porphyromonas* (Q)		0.045	0.674
1st tertile	Reference		
2nd tertile	0.424 (0.21; 0.85)		
3rd tertile	0.817 (0.44; 1.53)		
*Murdochiella* (Q)		0.006	0.540
1st tertile	Reference		
2nd tertile	0.253 (0.11; 0.59)		
3rd tertile	0.748 (0.37; 1.50)		
*Peptostreptococcus* *** (Q)		0.025	0.666
1st tertile	Reference		
2nd tertile	0.510 (0.25; 1.04)		
3rd tertile	1.348 (0.72; 2.53)		
*Allisonella* (Q)		0.036	0.666
1st tertile	Reference		
2nd tertile	2.204 (1.21; 4.02)		
3rd tertile	1.539 (0.85; 2.80)		
*Megamonas* (B)		0.032	0.666
1st tertile	Reference		
2nd tertile	1.794 (0.91; 3.55)		
3rd tertile	2.408 (1.25; 4.65)		
Exposure: ultra-processed foods consumption at 11 years			
*Bacteroides* *** (Q)		0.015	0.428
1st tertile	Reference		
2nd tertile	-0.254 (-0.50; -0.01)		
3rd tertile	-0.354 (-0.60; -0.11)		
*Roseburia* (B)		0.019	0.428
1st tertile	Reference		
2nd tertile	0.245 (-0.02; 0.51)		
3rd tertile	-0.123 (-0.40; 0.15)		
*Butyricimonas* (B)		0.010	0.428
1st tertile	Reference		
2nd tertile	1.861 (0.98; 3.54)		
3rd tertile	2.925 (1.44; 5.96)		
*Peptostreptococcus* *** (B)		0.010	0.428
1st tertile	Reference		
2nd tertile	0.704 (0.34; 1.45)		
3rd tertile	1.919 (0.99; 3.72)		
*Pseudoflavonifractor* (B)		0.030	0.428
1st tertile	Reference		
2nd tertile	0.547 (0.28; 1.08)		
3rd tertile	0.371 (0.17; 0.80)		
Exposure: ultra-processed foods consumption at 12 years			
*Ruminococcus2* (B)		0.047	0.495
1st tertile	Reference		
2nd tertile	0.167 (-0,10; 0.43)		
3rd tertile	0.341 (0.07; 0.61)		
*Methanobrevibacter* (B)		0.009	0.495
1st tertile	Reference		
2nd tertile	2.256 (1.25; 4.07)		
3rd tertile	2.149 (1.86; 3.89)		
*Senegalimassilia* (B)		0.032	0.495
1st tertile	Reference		
2nd tertile	1.037 (0.57; 1.88)		
3rd tertile	2.080 (1.12; 3.85)		

95%CI: 95% confidence interval; B: binary model (assessed using binary logistic regression, with results expressed as OR); FDR: false discovery rate; OR: odds ratio; Q: quantitative model (assessed using linear regression, with results expressed as β coefficients).

* Model adjusted for perinatal variables (gestational age, type of delivery, birth weight, parity, total family income, sex, skin color), breastfeeding duration, other sources of consumption in the NOVA classification, body mass index and use of antibiotics six months before the interview at age 12;

** p-value for the test Parm;

*** Genus nominally associates in more than one follow-up assessment.

## Discussion

This study used a population-based birth subsample to examine how ultra-processed food consumption in childhood and early adolescence affects the gut microbiota of Brazilian adolescents. We found no significant associations between ultra-processed food consumption and alpha diversity after correction, nor strong evidence linking it to beta diversity. However, nominal associations were observed between ultra-processed food consumption and abundances of *Actinobacteria*, *Proteobacteria*, *Bacteroides*, and *Peptostreptococcus* in various occasions.

The impact of ultra-processed food consumption on gut microbiome diversity is actively debated in the literature. Regarding alpha diversity, our research revealed an association between ultra-processed food and elevated mean alpha diversity indices (Chao1 and Shannon Entropy) prior to adjusting for covariates at ages 11 and 12. However, subsequent incorporation of covariates, as well as antibiotic usage, substantially attenuated the strength of this association. The multiple testing corrections rendered the effects on alpha diversity measures non-significant. The lack of association between ultra-processed food consumption and alpha diversity is in accordance to previous studies ^18^ ^,^ ^45^ ^,^ ^46^. These studies were carried out with women from 18 to 40 years old ^18^, institutionalized older men ^45^ and men and women aged from 31 to 50 years ^46^, with a cross-sectional design, and only variables such as race, BMI and age were used. In contrast, other studies have reported a negative association between high ultra-processed food consumption and alpha diversity ^20^ ^,^ ^22^ ^,^ ^47^, showing reduced diversity among individuals consuming unhealthy foods like fried products, sugary drinks, processed meats, and ready-made meals, compared to those who consume fresh fruits and fish. However, these studies are cross-sectional, focused on adults, and adjusted for only a limited number of confounders, such as sex ^20^ ^,^ ^22^, BMI ^20^ ^,^ ^22^, age, smoking, physical activity ^20^ and energy intake ^22^. Despite this, one study found a negative correlation between consumption of fried products and sugary drinks and alpha diversity indices after adjusting for diet, drugs, smoking, and diseases ^47^. Hence, the association between ultra-processed food and gut microbiota diversity remains a subject of ongoing inquiry in the scientific literature.

For beta diversity, which reflects differences in species composition among samples, the significance observed at 11 and 12 years of age was accompanied by overlapping patterns among tertiles of ultra-processed food consumption, suggesting no clear distinction in gut microbiota composition across these tertiles. The small R^2^ value further indicates a limited explanatory power, suggesting that other factors may play a larger role in influencing microbial composition. This finding aligns with previous observational studies in Spanish adults (n = 359) ^22^, healthy French adults (n = 862) ^20^ and older subjects aged 55-75 years (n = 645) ^24^, which reported no differences in beta diversity between ultra-processed food consumption groups.

No associations remained statistically significant after correction to analysis of relative abundance, but nominal associations were identified between ultra-processed food consumption, as well as the *Actinobacteria* phylum and the *Bacteroides* genus. While the literature lacks extensive studies investigating the abundance of *Actinobacteria* in relation to ultra-processed food consumption, our findings are supported by an observational study ^22^. Following sex-stratified analyses, researchers noted an increase in taxa abundance at both class and phylum levels *Actinobacteria* among individuals with the highest daily ultra-processed food consumption, as assessed via FFQ. Additionally, high abundance of this phylum has been linked to a high-fat, low-fiber diet in another study, which aimed to investigate the relationship between diet and enterotypes in 98 individuals ^4^. Finally, an animal model study ^48^ involving young female mice (n = 16) fed a ultra-processed food diet sourced from a fast food chain for six weeks demonstrated a higher abundance of *Actinobacteria* compared to controls during postnatal development.

Among the genera, the most consistent finding was the association between ultra-processed food consumption and *Bacteroides* at 6 and 11 years. Our results showed a statistically significant reduction in *Bacteroides* abundance among individuals in the intermediate (second tertile) and highest ultra-processed food consumption groups. These findings align with a cross-sectional study of 59 women (mean age = 28.0 ± 6.6 years) ^18^, which found a negative correlation between *Bacteroides* abundance and ultra-processed food consumption based on the NOVA classification. However, this contrasts with another cross-sectional study in Spanish adults, where a higher abundance at this phylum was observed in men in the highest tertile of ultra-processed food consumption ^22^. The *Bacteroides* genus, composed primarily of gram-negative bacteria, is a key component of the human microbiota, maintaining microbial balance ^49^. These bacteria help digest complex polysaccharides, such as dietary fibers, potentially reducing nutrient availability for other bacteria and influencing microbial competition and diversity ^49^. *Bacteroides* has been linked to intestinal inflammation by modulating pro-inflammatory cells ^50^, contributing to conditions like inflammatory bowel disease and metabolic disorders, including obesity and type 2 diabetes ^51^ ^,^ ^52^.

Given that ultra-processed foods are typically high in energy, added sugars, salt, saturated and trans fats, and low in fiber, protein, and micronutrients ^42^, they may induce unfavorable shifts in microbiota composition, promoting the growth of inflammatory bacteria associated with conditions such as type 2 diabetes, cardiovascular diseases, and metabolic disorders ^12^ ^,^ ^53^. This suggests that the impact of ultra-processed food consumption on *Actinobacteria* and *Bacteroides* abundance is complex and likely modulated by overall diet quality, nutrient intake, and microbiome health. Further research is needed to clarify these relationships. Since our results were not significant after multiple comparisons, further speculation on this association would be premature.

Notably, our study differs from previous ones regarding population origin, design, and age demographics. The microbiome during childhood may differ significantly from its composition during adulthood, particularly concerning diversity and composition ^54^. However, the literature in this age range is scarce. In a cross-sectional study conducted with 30 children aged between 1 and 6, the authors found that European children had lower richness (Chao1 index) compared to children living in rural areas of Africa, whose habit is to consume foods rich in fiber ^2^, revealing the need for more studies covering childhood and adolescence.

Finally, not only did we use a different population origin, but also, we adjusted our statistical models for several maternal and child variables that were not included in previous models, even with clear evidence of association with microbiome ^55^. Notably, most existing research has been conducted in high-income countries ^2^ ^,^ ^22^ ^,^ ^24^, which may differ in factors influencing ultra-processed food consumption and gut microbiota from low- and middle-income countries ^56^. Behavioral and economic factors in these different contexts could lead to varying confounding structures. For example, in high-income countries, higher ultra-processed food consumption is often linked to higher BMI ^57^ and lower socioeconomic status ^58^. Conversely, in our study, higher ultra-processed food consumption was more common among adolescents from lower socioeconomic backgrounds, but with lower BMI (Supplementary Material - Table S2; https://cadernos.ensp.fiocruz.br/static//arquivo/suppl-e00094424_9468.pdf). The inconsistency between our findings and those previously reported might be due to differences in adjustment or even potential differential confounding structures across populations. Therefore, the inconsistency in our findings underscores the need for further and larger longitudinal studies in diverse populations evaluating ultra-processed food consumption and microbiome composition with adjustment for potential confounders (including the ones related to birth and perinatal well-being).

To the best of our knowledge, this is the first study to investigate the longitudinal relationship between ultra-processed food consumption and gut microbiota in adolescents. This study has several strengths. The first is its longitudinal methodology with data from multiple follow-ups since birth, enhancing the reliability of our findings and reducing reverse causality biases. Further, we thoroughly adjusted various potential confounders, including birth, perinatal-related, socioeconomic, anthropometric, and dietary factors. The sample size is also higher than most published studies investigating determinants of the microbiota ^2^ ^,^ ^22^ ^,^ ^23^. Lastly, our research addresses a significant gap in the current literature by focusing on a population from a low- and middle-income countries, a group that has been comparatively underrepresented in existing studies.

However, our study has some limitations. The first is relying on FFQ for the collection of habitual dietary information. Although FFQs are advantageous due to their cost-benefit ratio, being practical and capable of providing quantitative consumption estimates, and widely used in epidemiological studies ^59^, they are susceptible to measurement errors, since they depend on the respondents’ recall ability. Additionally, while our study benefits from a larger sample size compared to most research in this field, it is conceivable that even larger sample sizes might be required to adequately power the analysis of some associations. Furthermore, despite our efforts to account for several potential confounding factors in the models, residual confounding may still introduce bias into our results. Literature indicates that gut microbiota is influenced by various factors, including environmental conditions like exposure to pollutants and the presence of household pets, which can affect the abundance and presence of certain taxa. Additionally, given the limited research on gut microbiota in adolescents, age-related factors such as hormonal changes during adolescence, sleep patterns, lifestyle, risk behaviors, and stress may also play a significant role.

Although adjustments for multiple testing revealed no statistically significant associations in diversity and relative abundance metrics, our results indicate that ultra-processed food consumption may influence gut microbiome composition. Specifically, ultra-processed food intake appears to affect the relative abundance of *Actinobacteria* and *Bacteroides* in adolescents. The research underscores the need for future studies with larger sample sizes, from varied geographic and demographic regions, to further explore how ultra-processed foods affect gut microbiota diversity and abundance across different age groups and settings. Mainly due to the role that the frequent consumption of such foods can have as atherogenic triggers in processes such as dyslipidemia, hypertension and obesity, which represent an emerging public health issue.
