# Supplementary material for: Exploring the relationship between ultra-processed food consumption and gut microbiota at school age in a Brazilian birth cohort
Source: Cad Saude Publica. 2025 Mar 31;41(2):e00094424. doi: 10.1590/0102-311XEN094424 (PMC11960759; doi:10.1590/0102-311XEN094424)
Supplement: Supplementary file 1 [file 1678-4464-csp-41-02-EN094424-s.pdf]

SUPPLEMENTARY MATERIAL

Figure S1 Flowchart of the microbiome substudy.

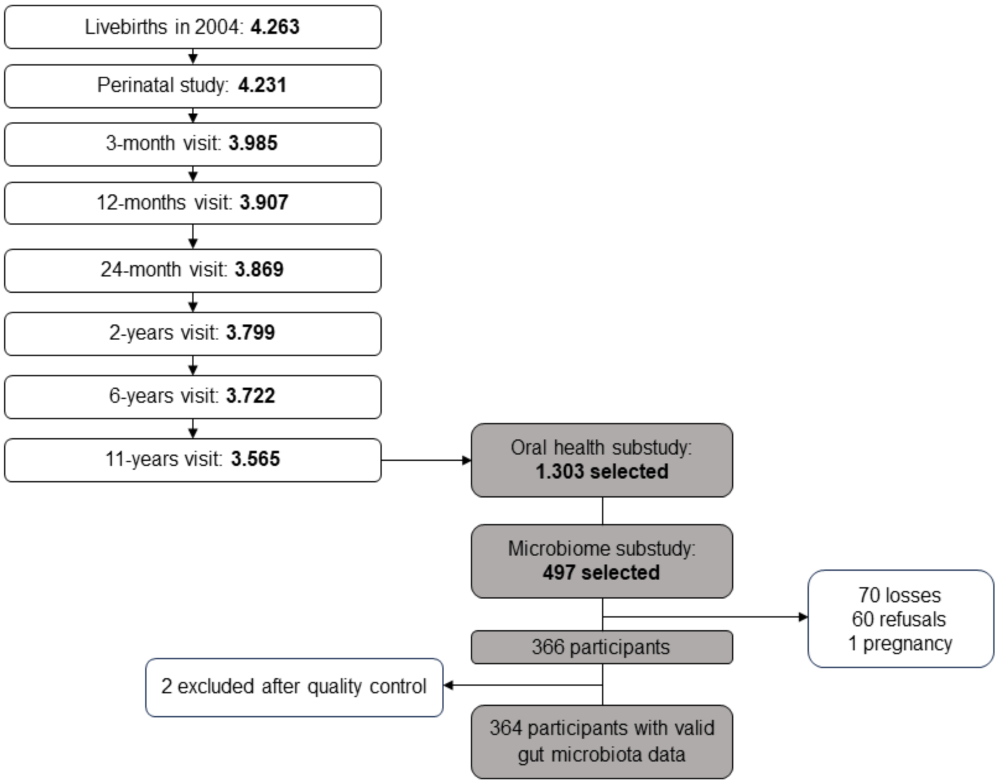

Box S1 Food items included in the FFQ.

GROUP 1 – UNPROCESSED OR MINIMALLY PROCESSED FOODS	
6 years	11 and 12 years
Rice; spaghetti; cakes; potato; cassava; bean; lettuce; tomato; pumpkin; cabbage; broccoli; raw carrot; cooked carrot; raw beet; cooked beet; chayote; orange; banana; apple; papaya; tangerine; pear; natural juice; whole milk; skimmed milk; beef with bone; boneless beef; liver; chicken; fish; eggs; coffee.	Rice; spaghetti; homemade cake; boiled potatoes; fried potato, *polenta* or cassava; cow milk; soy milk; orange or tangerine; banana; papaya; apple or pear; watermelon; melon; pineapple; avocado; mango; strawberry; grape; peach; guava; natural juice; lettuce; tomato; collard greens; cabbage; chayote; pumpkin; natural cucumber; green beans; carrot; beet; cauliflower; pepper; onion; garlic; bean; lentil, pea or chickpeas; deep-fried and salted (*quibe*, *pastel*); coffee; tea; chestnut, walnut or almond; homemade pudding; beef; pork; chicken; fish; shrimp; viscera (gizzard, liver, heart, kidney); egg; popcorn; lasagna, gnocchi or ravioli.
**GROUP 2 – PROCESSED CULINARY INGREDIENTS**	
6 years	11 and 12 years
Sugar	Sugar
**GROUP 3 – PROCESSED FOOD**	
6 years	11 and 12 years
Bread; cheese; jelly or fruit jam.	Beer; wine; yellow cheese; white cheese; bacon; canned fish; canned food (peas, corn); whole grain bread; white bread; jelly or fruit jam.
**GROUP 4 – ULTRA-PROCESSED FOOD**	
Sweet cookies; salty cracker; yogurt or milk drink; ham; mortadella; sausage; butter or margarine; mayonnaise; candies, lollipop or chewing gum; chocolate bar or bonbon; ice cream or popsicle; chocolate powder; sugar-sweetened beverages; artificially sweetened beverages (light, diet or zero); artificial juice (powder or box); salty snacks; sandwich cookies; gelatin.	Sweet cookie; salty cookie; granola; breakfast cereals; cereal bar; instant noodles; yogurt or milk drink; cream cheese; burger or nuggets; mortadella or ham or salami; sausages; butter/margarine; mayonnaise; ice cream or popsicle; lollipop and chewing gum; chocolate powder; chocolate bar; sugar-sweetened beverages; artificial juice; cachaça or whiskey or vodka or other drinks; chips and snacks; pizza; cheeseburger or hot dog.

Table S1

UPF Consumption 6 anos						
**Exposure:**	**Model**	**Crude model**		**Adjusted model#**		**FDR**
**UPF consumption**		**Beta/OR (IC95%)**	**P value**	**Beta/OR (IC95%)**	**P value**	**P value**
		**Genus**				
G_Bacteroides	Q		**0.011***		**0.037***	0.666
1st Tertile		Ref		Ref		
2st Tertile		-0.222 (-0.47; 0.03)	0.083	-0.132 (-0.37; 0.11)	0.280	
3st Tertile		-0.386 (-0.64; -0.14)	**0.003**	-0.315 (-0.56; -0.07)	**0.011**	
G_Parabacteroides	Q		0.107*		0.510*	0.988
1st Tertile		Ref		Ref		
2st Tertile		0.123 (-0.13; 0.38)	0.340	-0.138 (-0.39; 0.11)	0.282	
3st Tertile		0.216 (-0.04; 0.47)	0.096	-0.116 (-0.37;0.13)	0.362	
G_Prevotella	Q		0.247*		0.308*	0.988
1st Tertile		Ref		Ref		
2st Tertile		0.123 (-0.13; 0.38)	0.340	0.126 (-0.12; 0.37)	0.322	
3st Tertile		0.216 (-0.04; 0.47)	0.096	0.191 (-0.06; 0.44)	0.131	
G_Alistipes	Q		0.354*		0.506*	0.988
1st Tertile		Ref		Ref		
2st Tertile		-0.159 (-0.41; 0.09)	0.218	-0.138 (-0.39; 0.11)	0.281	
3st Tertile		-0.163 (-0.42; 0.09)	0.207	-0.118 (-0.36; 0.13)	0.355	
G_Clostridium_Sensu_Stricto	Q		0.639*		0.519*	0.988
1st Tertile		Ref		Ref		
2st Tertile		-0.017 (-0.27; 0.24)	0.899	-0.138 (-0.41; 0.13)	0.312	
3st Tertile		0.098 (-0.16; 0.36)	0.455	-0.006 (-0.27; 0.26)	0.965	
G_Anaerostipes	Q		0.389*		0.769*	0.988
1st Tertile		Ref		Ref		
2st Tertile		-0.167 (-0.42; 0.09)	0.195	-0.075 (-0.33; 0.18)	0.556	
3st Tertile		-0.135 (-0.39; 0.12)	0.297	0.009 (-0.24; 0.26)	0.942	
G_Blautia	Q		0.739*		0.947*	0.988
1st Tertile		Ref		Ref		
2st Tertile		-0.085 (-0.34; 0.17)	0.509	-0.016 (-0.27; 0.24)	0.901	
3st Tertile		-0.088 (-0.34; 0.16)	0.493	0.026 (-0.23; 0.28)	0.839	
G_Clostridium_xlva	Q		0.837*		0.675*	0.988
1st Tertile		Ref		Ref		
2st Tertile		0.041 (-0.21; 0.30)	0.752	-0.018 (-0.29; 0.25)	0.895	
3st Tertile		-0.036 (-0.29; 0.22)	0.779	-0.114 (-0.39; 0.16)	0.410	
G_Coprococcus	Q		0.667*		0.628*	0.988
1st Tertile		Ref		Ref		
2st Tertile		-0.027 (-0.28; 0.23)	0.835	-0.053 (-0.32; 0.22)	0.697	
3st Tertile		0.085 (-0.17; 0.34)	0.513	0.078 (-0.19; 0.35)	0.569	
G_Dorea	Q		0.831*		0.835*	0.988
1st Tertile		Ref		Ref		
2st Tertile		0.068 (-0.19; 0.33)	0.601	0.053 (-0.22; 0.33)	0.704	
3st Tertile		0.068 (-0.19; 0.33)	0.601	0.083 (-0.19; 0.36)	0.554	
G_Fusicatenibacter	Q		0.388*		0.803*	0.988
1st Tertile		Ref		Ref		
2st Tertile		-0.147 (-0.40; 0.11)	0.256	-0.086 (-0.34; 0.17)	0.513	
3st Tertile		-0.160 (-0.41; 0.09)	0.216	-0.054 (-0.31; 0.20)	0.679	
G_Roseburia	Q		0.577*		0.905*	0.988
1st Tertile		Ref		Ref		
2st Tertile		-0.032 (-0.29; 0.22)	0.805	0.032 (-0.24; 0.30)	0.812	
3st Tertile		-0.132 (-0.39; 0.13)	0.315	-0.028 (-0.29; 0.24)	0.836	
G_Ruminococcus2	Q		0.399*		0.335*	0.988
1st Tertile		Ref		Ref		
2st Tertile		-0.099 (-0.36; 0.16)	0.447	-0.061 (-0.33; 0.21)	0.656	
3st Tertile		0.077 (-0.17; 0.33)	0.554	0.137 (-0.13; 0.41)	0.317	
G_Intestinibacter	Q		0.621*		0.367*	0.988
1st Tertile		Ref		Ref		
2st Tertile		-0.087 (-0.34; 0.17)	0.501	-0.117 (-0.38; 0.15)	0.388	
3st Tertile		-0.123 (-0.38; 0.13)	0.343	-0.190 (-045; 0.07)	0.161	
G_Romboutsia	Q		0.870*		0.679*	0.988
1st Tertile		Ref		Ref		
2st Tertile		-0.050 (-0.31; 0.21)	0.700	-0.100 (-0.36; 0.16)	0.454	
3st Tertile		0.015 (-0.24; 0.27)	0.906	0.003 (-0.26; 0.27)	0.980	
G_Clostridium_IV	Q		0.301*		0.428*	0.988
1st Tertile		Ref		Ref		

2st Tertile		-0.194 (-0.45; 0.06)	0.136	-0.162 (-0.43; 0.11)	0.237	
3st Tertile		-0.142 (-0.39; 0.11)	0.276	-0.145 (-0.41; 0.12)	0.288	
G_Faecalibacterium	Q		0.864*		0.603*	0.988
1st Tertile		Ref		Ref		
2st Tertile		-0.032 (-0.29; 0.22)	0.804	0.035 (-0.22; 0.29)	0.790	
3st Tertile		0.037 (-0.22; 0.29)	0.770	0.127 (-0.13; 0.38)	0.331	
G_Gemmiger	Q		0.116*		0.226*	0.988
1st Tertile		Ref		Ref		
2st Tertile		-0.268 (-0.52; -0.02)	**0.038**	-0.194 (-0.46; 0.07)	0.147	
3st Tertile		-0.128 (-0.38; 0.12)	0.319	0.011 (-0.25; 0.27)	0.935	
G_Oscillibacter	Q		0.122*		0.305*	0.988
1st Tertile		Ref		Ref		
2st Tertile		-0.100 (-0.36; 0.16)	0.443	-0.009 (-0.27; 0.26)	0.944	
3st Tertile		-0.266 (-0.52; -0.01)	**0.042**	-0.184 (-0.45; 0.08)	0.172	
G_Ruminococcus	Q		0.570*		0.749*	0.988
1st Tertile		Ref		Ref		
2st Tertile		-0.137 (-0.39; 0.12)	0.290	-0.102 (-0.37; 0.16)	0.448	
3st Tertile		-0.072 (-0.33; 0.18)	0.578	-0.050 (-0.31; 0.21)	0.709	
G_Lachnospiracea_incertae_sedis	Q		0.194*		0.092*	0.685
1st Tertile		Ref		Ref		
2st Tertile		0.152 (-0.11; 0.41)	0.244	0.272 (0.01; 0.53)	**0.040**	
3st Tertile		-0.081 (-0.34; 0.17)	0.535	1.316 (0.74; 2.34)	0.350	
G_Erysipelotrichaceae_incertae_sedis	B		0.097*		0.157*	0.923
1st Tertile		Ref		Ref		
2st Tertile		0.635 (037; 1.07)	0.087	0.607 (034; 1.08)	0.088	
3st Tertile		0.589 (0.35; 0.99)	**0.048**	0.627 (0.35; 1.11)	0.110	
G_Methanobrevibacter	B		0.129*		0.306*	0.988
1st Tertile		Ref		Ref		
2st Tertile		1.235 (0.73 2.08)	0.427	1.113 (0.63; 1.97)	0.714	
3st Tertile		1.733 (1.01; 2.96)	**0.044**	1.561 (0.87; 2.81)	0.137	
G_Methanosphaera	B		0.721*		0.854*	0.988
1st Tertile		Ref		Ref		
2st Tertile		1.295 (0.69; 2.44)	0.423	1.224 (0.60; 2.48)	0.575	
3st Tertile		1.114 (0.58; 2.12)	0.742	1.113 (0.54; 2.29)	0.771	
G_Actinomyces	B		0.967*		0.966*	0.988
1st Tertile		Ref		Ref		
2st Tertile		0.967 (0.58; 1.61)	0.896	0.936 (0.54; 1.62)	0.813	
3st Tertile		1.035 (0.62; 1.72)	0.896	0.995 (0.58; 1.72)	0.985	
G_Rothia	B		0.173*		0.054*	0.685
1st Tertile		Ref		Ref		
2st Tertile		1.123 (0.58; 2.19)	0.734	1.165 (0.56; 2.41)	0.678	
3st Tertile		1.746 (0.93; 3.28)	0.084	2.193 (1.09; 4.39)	**0.027**	
G_Bifidobacterium	B		0.681*		0.656*	0.988
1st Tertile		Ref		Ref		
2st Tertile		0.767 (0.25; 2.19)	0.583	0.544 (0.15; 1.99)	0.358	
3st Tertile		1.211 (0.36; 4.08)	0.758	0.711 (0.18; 2.83)	0.628	
G_Collinsela	B		**0.046***		0.076*	0.685
1st Tertile		Ref		Ref		
2st Tertile		3.226 (1.01; 10.31)	**0.048**	4.51 (1.09; 18.70)	**0.038**	
3st Tertile		3.226 (1.01; 10.31)	**0.048**	2.74 (0.73; 10.26)	0.134	
G_Eggerthella	B		**0.008***		0.078*	0.685
1st Tertile		Ref		Ref		
2st Tertile		0.559 (0.33; 0.94)	**0.027**	0.643 (0.36; 1.14)	0.132	
3st Tertile		0.453 (0.29; 0.76)	**0.003**	0.525 (0.30; 0.93)	**0.028**	
G_Gordonibacter	B		0.592*		0.828*	0.988
1st Tertile		Ref		Ref		
2st Tertile		0.772 (0.43; 1.37)	0.380	0.831 (0.44; 1.59)	0.577	
3st Tertile		0.772 (0.43; 1.37)	0.380	0.850 (0.45; 1.62)	0.623	
G_Olsenella	B		0.485*		0.410*	0.988
1st Tertile		Ref		Ref		
2st Tertile		0.900 (0.54; 1.51)	0.691	0.680 (0.38; 1.22)	0.195	
3st Tertile		1.228 (0.73; 2.05)	0.433	0.898 (0.51; 1.59)	0.712	
G_Senegalimassilia	B		0.282*		0.880*	0.988
1st Tertile		Ref		Ref		
2st Tertile		1.312 (0.79; 2.19)	0.298	1.168 (0.64; 2.13)	0.612	
3st Tertile		1.506 (0.90; 2.52)	0.118	1.083 (0.600; 1.96)	0.791	
G_Slackia	B		0.733*		0.858*	0.988
1st Tertile		Ref		Ref		
2st Tertile		1.000 (0.59; 1.70)	1.000	0.839 (0.45; 1.56)	0.581	
3st Tertile		1.207 (0.70; 1.70)	0.493	0.909 (0.49; 1.69)	0.763	

G_Barnesiella	B		0.415*		0.372*	0.988
1st Tertile		Ref		Ref		
2st Tertile		0.681 (0.38; 1.21)	0.191	0.645 (0.35; 1.20)	0.167	
3st Tertile		0.769 (0.43; 1.38)	0.375	0.736 (0.39; 1.38)	0.337	
G_Butyricimonas	B		0.466*		0.922*	0.988
1st Tertile		Ref		Ref		
2st Tertile		1.201 (0.66; 2.17)	0.546	0.964 (0.50; 1.85)	0.912	
3st Tertile		1.472 (0.80; 2.72)	0.216	1.103 (0.57; 2.14)	0.773	
G_Coprobacter	B		0.653*		0.709*	0.988
1st Tertile		Ref		Ref		
2st Tertile		0.865 (0.41; 1.83)	0.704	0.958 (0.43; 2.13)	0.917	
3st Tertile		1.212 (0.60; 2.45)	0.592	1.292 (0.60; 2.78)	0.511	
G_Odoribacter	B		0.440*		0.497*	0.988
1st Tertile		Ref		Ref		
2st Tertile		0.521 (0.19; 1.46)	0.215	0.599 (0.20; 1.77)	0.354	
3st Tertile		0.579 (0.20; 1.65)	0.305	0.529 (0.18; 1.57)	0.252	
G_Porphyromonas	B		0.104*		**0.045***	0.674
1st Tertile		Ref		Ref		
2st Tertile		0.505 (0.26; 0.97)	0.039	0.424 (0.21; 0.85)	**0.015**	
3st Tertile		0.873 (0.48; 1.57)	0.652	0.818 (0.44; 1.53)	0.528	
G_Paraprevotella	B		0.702*		0.492*	0.988
1st Tertile		Ref		Ref		
2st Tertile		1.228 (0.75; 2.05)	0.433	0.979 (0.56; 1.72)	0.942	
3st Tertile		1.035 (0.62; 1.73)	0.895	0.735 (0.42; 1.29)	0.285	
G_Lactobacillus	B		0.470*		0.903*	0.988
1st Tertile		Ref		Ref		
2st Tertile		1.000 (0.60; 1.68)	1.000	0.859 (0.51; 1.58)	0.704	
3st Tertile		1.333 (0.79; 2.26)	0.284	1.006 (0.57; 1.78)	0.985	
G_Lactococcus	B		0.348*		0.099*	0.685
1st Tertile		Ref		Ref		
2st Tertile		0.571 (0.26; 1.27)	0.169	0.409 (0.17; 1.01)	0.052	
3st Tertile		0.935 (0.46; 1.92)	0.855	1.012 (0.46; 2.24)	0.976	
G_Streptococcus	B		0.510*		0.447*	0.988
1st Tertile		Ref		Ref		
2st Tertile		0.826 (0.25; 2.78)	0.758	1.130 (0.28; 4.49)	0.862	
3st Tertile		0.536 (0.17; 1.65)	0.277	1.525 (0.56; 2.27)	0.742	
G_Christensenella	B		0.545*		0.437*	0.988
1st Tertile		Ref		Ref		
2st Tertile		1.000 (0.53; 1.90)	1.000	1.125 (0.56; 2.27)	0.742	
3st Tertile		1.346 (0.72; 2.50)	0.347	1.525 (0.78; 2.99)	0.219	
G_Anaerococcus	B		0.847*		0.978*	0.989
1st Tertile		Ref		Ref		
2st Tertile		1.077 (0.51; 2.29)	0.847	1.083 (0.49; 2.40)	0.845	
3st Tertile		1.2236 (0.59; 2.59)	0.574	1.070 (0.49; 2.36)	0.867	
G_Ezakiella	B		0.210*		0.280*	0.988
1st Tertile		Ref		Ref		
2st Tertile		0.482 (0.21; 1.09)	0.079	0.498 (0.21; 1.17)	0.111	
3st Tertile		0.817 (0.40; 1.68)	0.583	0.794 (0.37; 1.72)	0.558	
G_Murdochiella	B		**0.002***		**0.006***	0.540
1st Tertile		Ref		Ref		
2st Tertile		0.242 (0.11; 0.54)	**<0.001**	0.253 (0.11; 0.59)	**0.002**	
3st Tertile		0.672 (0.36; .25)	0.210	0.748 (0.37; 1.50)	0.414	
G_Parvimonas	B		0.276*		0.349*	0.988
1st Tertile		Ref		Ref		
2st Tertile		0.587 (0.28; 1.21)	0.150	0.616 (0.28; 1.35)	0.225	
3st Tertile		0.635 (0.31; 1.30)	0.213	0.615 (0.28; 1.33)	0.217	
G_Mogibacterium	B		0.138*		0.164*	0.923
1st Tertile		Ref		Ref		
2st Tertile		0.820 (0.44; 1.52)	0.530	0.649 (0.33; 1.28)	0.214	
3st Tertile		1.468 (0.83; 2.61)	0.191	1.219 (0.65; 2.30)	0.541	
G_Anaerofustis	B		0.443*		0.719*	0.988
1st Tertile		Ref		Ref		
2st Tertile		0.590 (0.26; 1.36)	0.216	0.699 (0.28; 1.74)	0.442	
3st Tertile		0.722 (0.33; 1.60)	0.422	0.774 (0.32; 1.87)	0.570	
G_Eubacterium	B		**0.013***		0.067*	0.685
1st Tertile		Ref		Ref		
2st Tertile		1.546 (0.81; 2.97)	0.191	1.296 (0.63; 2.67)	0.482	
3st Tertile		3.204 (1.47; 6.97)	**0.003**	1.67 (1.16; 6.14)	**0.020**	
G_Butyrivibrio	B		0.737*		0.464*	0.988
1st Tertile		Ref		Ref		

2st Tertile		1.200 (0.71; 2.04)	0.500	1.180 (0.65; 2.13)	0.583	
3st Tertile		1.000 (0.58; 1.71)	1.000	0.812 (0.45; 1.48)	0.494	
G_Clostridium_XLVb	B		0.770*		0.783*	0.988
1st Tertile		Ref		Ref		
2st Tertile		0.785 (0.30; 2.06)	0.624	0.806 (0.28; 2.30)	0.687	
3st Tertile		0.707 (0.27; 1.83)	0.475	0.687 (0.24; 1.97)	0.484	
G_Eisenbergiella	B		0.131*		0.752*	0.988
1st Tertile		Ref		Ref		
2st Tertile		0.687 (0.41; 1.15)	0.152	0.752 (0.42; 1.35)	0.340	
3st Tertile		0.599 (0.36; 1.00)	0.051	0.642 (0.36; 1.15)	0.136	
G_Howardella	B		**0.045***		0.321*	0.988
1st Tertile		Ref		Ref		
2st Tertile		1.993 (1.15 3.45)	**0.014**	1.786 (0.97; 3.30)	0.064	
3st Tertile		1.610 (0.92; 2.80)	0.093	1.323 (0.71; 2.45)	0.374	
G_Peptococcus	B		0.241*		0.492*	0.988
1st Tertile		Ref		Ref		
2st Tertile		1.652 (0.91; 3.00)	0.100	1.474 (0.77; 2.82)	0.241	
3st Tertile		1.457 (0.80; 2.67)	0.222	1.316 (0.68; 2.53)	0.412	
G_Peptoniphilus	B		0.394*		0.685*	0.988
1st Tertile		Ref		Ref		
2st Tertile		0.658 (0.36; 1.20)	0.172	0.749 (0.39; 1.44)	0.387	
3st Tertile		0.839 (0.47; 1.50)	0.554	0.856 (0.45; 1.62)	0.632	
G_Clostridium_XI	B		0.677*		0.945*	0.988
1st Tertile		Ref		Ref		
2st Tertile		1.330 (0.68; 2.59)	0.400	1.088 (0.53; 2.30)	0.818	
3st Tertile		1.261 (0.5; 2.46)	0.497	1.128 (0.55; 2.31)	0.743	
G_Peptostreptococcus	B		**0.040***		**0.025***	0.666
1st Tertile		Ref	0.054	Ref		
2st Tertile		0.528 (0.28; 1.01)	0.558	0.510 (0.25; 1.04)	0.064	
3st Tertile		1.187 (0.67; 2.11)		1.348 (0.72; 2.54)	0.355	
G_Terrisporobacter	B		0.732*		0.416*	0.988
1st Tertile		Ref		Ref		
2st Tertile		0.833 0.46; 1.51)	0.546	0.658 (0.35; 1.25)	0.203	
3st Tertile		1.044 (0.59; 1.86)	0.883	0.914 (0.49; 1.70)	0.777	
G_Anaerofilum	B		0.424*		0.233*	0.988
1st Tertile		Ref		Ref		
2st Tertile		0.815 (0.49; 1.36)	0.433	0.810 (0.46; 1.42)	0.461	
3st Tertile		1.145 (0.69; 1.91)	0.603	1.318 (0.76; 2.30)	0.331	
G_Anaerotruncus	B		0.474*		0.773*	0.988
1st Tertile		Ref		Ref		
2st Tertile		0.648 (0.32; 1.30)	0.222	0.761 (0.36; 1.61)	0.474	
3st Tertile		0.769 (0.38; 1.57)	0.470	0.856 (0.39; 1.86)	0.694	
G_Flavonifractor	B		0.077*		0.222*	0.988
1st Tertile		Ref		Ref		
2st Tertile		0.484 (0.25; 0.92)	**0.027**	0.561 (0.27; 1.15)	0.113	
3st Tertile		0.743 (0.38; 1.46)	0.391	0.894 (0.42; .90)	0.771	
G_Intestinimonas	B		**0.036***		0.161*	0.923
1st Tertile		Ref		Ref		
2st Tertile		0.587 (0.35; 0.99)	**0.047**	0.662 (0.37; 1.17)	0.159	
3st Tertile		0.523 (0.31; 0.89)	**0.017**	0.590 (0.33; 1.05)	0.072	
G_Pseudoflavonifractor	B		0.083*		0.662*	0.988
1st Tertile		Ref		Ref		
2st Tertile		0.658 (0.36; 1.20)	0.172	0.859 (0.43; 1.72)	0.668	
3st Tertile		0.494 (0.26; 0.93)	**0.030**	0.717 (035; 1.47)	0.364	
G_Catenibacterium	B		0.643*		0.748*	0.988
1st Tertile		Ref		Ref		
2st Tertile		1.269 (0.76; 2.12)	0.362	0.922 (0.50; 1.70)	0.795	
3st Tertile		1.186 (0.71; 1.98)	0.514	0.794 (0.43; 1.45)	0.454	
G_Clostridium_XVIII	B		0.106*		0.210*	0.988
1st Tertile		Ref		Ref		
2st Tertile		0.494 (0.23; 1.05)	0.067	0.500 (0.22; 1.12)	0.092	
3st Tertile		0.468 (0.22; 0.99)	0.047	0.550 (0.24; 1.24)	0.151	
G_Coprobacillus	B		0.583*		0.657*	0.988
1st Tertile		Ref		Ref		
2st Tertile		1.186 (0.61; 2.30)	0.613	0.927 (0.45; 1.91)	0.839	
3st Tertile		0.824 (0.41; 1.67)	0.592	0.715 (0.34; 1.51)	0.380	
G_Holdemanella	B		**0.009***		0.085*	0.685
1st Tertile		Ref		Ref		
2st Tertile		1.155 (0.68; 1.95)	0.592	0.941 (0.51; 1.75)	0.848	
3st Tertile		2.342 (1.32; 4.14)	**0.003**	1.861 (0.97; 3.56)	0.060	

G_Holdemania	B		0.555*		0.537*	0.988
1st Tertile		Ref		Ref		
2st Tertile		0.761 (0.46; 1.27)	0.297	0.747 (0.43; 1.30)	0.305	
3st Tertile		0.934 (0.56; 1.56)	0.795	0.963 (0.55; 1.67)	0.894	
G_Turicibacter	B		0.506*		0.738*	0.988
1st Tertile		Ref		Ref		
2st Tertile		0.759 (0.37; 1.58)	0.459	0.798 (0.37; 1.74)	0.573	
3st Tertile		1.176 (0.53; 2.59)	0.687	1.080 (0,47; 2.47)	0.855	
G_Acidaminococcus	B		0.977*		0.963*	0.988
1st Tertile		Ref		Ref		
2st Tertile		1.069 (0.52; 2.19)	0.855	1.083 (0.50; 2.34)	0.840	
3st Tertile		1.000 (0.48; 2.07)	1.000	0.974 (0.45; 2.10)	0.948	
G_Phascolarctobacterium	B		0.811*		0.873*	0.988
1st Tertile		Ref		Ref		
2st Tertile		1.051 (0.57; 1,95)	0.874	0.951 (0.48; 1.88)	0.884	
3st Tertile		0.867 (0.47; 1.95)	0.644	0.843 (0.43; 1.64)	0.614	
G_Allisonella	B		**0.003***		**0.036***	0.666
1st Tertile		Ref		Ref		
2st Tertile		2.490 (1.45; 4.27)	**0.001**	2.204 (1.21; 4.02)	**0.010**	
3st Tertile		2.029 (1.18; 3.49)	**0.010**	1.539 (0.85; 2.80)	0.158	
G_Dialister	B		0.503*		0.712*	0.988
1st Tertile		Ref		Ref		
2st Tertile		1.036 (0.61; 1.75)	0.894	1.038 (0.59; 1.82)	0.898	
3st Tertile		1.341 (0.79; 2.28)	0.280	1.251 (0.71; 2.21)	0.440	
G_Megamonas	B		**0.038***		**0.032***	0.666
1st Tertile		Ref		Ref		
2st Tertile		1.437 (0.76; 2.71)	0.263	1.794 (0.91; 3.55)	0.093	
3st Tertile		2.194 (1.19; 4.04)	**0.012**	2.408 (1.25; 4.65)	**0.009**	
G_Megasphaera	B		0.883*		0.634*	0.988
1st Tertile		Ref		Ref		
2st Tertile		1.110 (0.59; 2.09)	0.746	1.042 (0.52; 2.09)	0.907	
3st Tertile		0.946 (0.49; 1.81)	0.868	0.754 (0.37; 1.56)	0.445	
G_Mitsuokella	B		0.892*		0.947*	0.988
1st Tertile		Ref		Ref		
2st Tertile		1.157 (0.63; 2.13)	0.640	0.939 (0.47; 1.88)	0.860	
3st Tertile		1.051 (0.57; 1.95)	0.874	0.890 (0.44; 1.78)	0.742	
G_Veillonella	B		0.463*		0.362*	0.988
1st Tertile		Ref		Ref		
2st Tertile		1.043 (0.59; 1.85)	0.884	1.222 (0.65; 2.30)	0.532	
3st Tertile		0.757 (0.44; 1.32)	0.325	0.783 (0.43; 1.44)	0.431	
G_Fusobacterium	B		0.489*		0.254*	0.988
1st Tertile		Ref		Ref		
2st Tertile		1.066 (0.53; 2.15)	0.858	1.152 (0.54; 2.44)	0.711	
3st Tertile		0.687 (0.32; 1.48)	0.337	0.585 (0.26; 1.33)	0.201	
G_Victivallis	B		0.952*		0.698*	0.988
1st Tertile		Ref		Ref		
2st Tertile		0.928 (0.54; 1.58)	0.786	0.795 (0.44; 1.42)	0.440	
3st Tertile		0.928 (0.54; 1.58)	0.786	0.814 (0.46; 1.45)	0.487	
G_Parasutterella	B		0.771*		0.905*	0.988
1st Tertile		Ref		Ref		
2st Tertile		0.815 (0.46; 1.43)	0.475	0.894 (0.49; 1.64)	0.718	
3st Tertile		0.919 (0.52; 1.62)	0.772	1.012 (0.55; 1.88)	0.968	
G_Sutterella	B		0.552*		0.209*	0.988
1st Tertile		Ref		Ref		
2st Tertile		1.000 (0.58; 1.71)	1.000	0.811 (0.45; 1.47)	0.490	
3st Tertile		0.776 (0.46; 1.32)	0.348	0.592 (0.33; 1;.06)	0.080	
G_Bilophila	B		0.842*		0.997*	0.997
1st Tertile		Ref		Ref		
2st Tertile		0.800 (0.37; 1.71)	0.564	1.018 (0.45; 2.33)	0.966	
3st Tertile		0.858 0.40; 1.85)	0.696	1.035 (0.44; 2.41)	0.937	
G_Desulfovibrio	B		0.895*		0.644*	0.988
1st Tertile		Ref		Ref		
2st Tertile		0.867 (0.47; 1.59)	0.644	0.771 (0.39; 1.52)	0.450	
3st Tertile		0.953 (0.52; 1.76)	0.876	0.742 (0.38; 1.46)	0.386	
G_Campylobacter	B		0.553*		0.564*	0.988
1st Tertile		Ref		Ref		
2st Tertile		1.101 (0.60; 2.02)	0.757	1.072 (0.56; 2.05)	0.834	
3st Tertile		1.374 (0.76; 2.48)	0.294	1.375 (0.74; 2.56)	0.316	
G_Succinivibrio	B		0.464*		0.840*	0.988
1st Tertile		Ref		Ref		

2st Tertile		1.694 (0.73; 3.91)	0.216	1.314 (0.53; 3.28)	0.558	
3st Tertile		1.337 (0.56; 3.18)	0.511	1.206 (0.47; 3.08)	0.694	
G_Escherichia/Shigella	B		0.747*		0.822*	0.988
1st Tertile		Ref		Ref		
2st Tertile		1.318 (63; 2.74)	0.459	1.206 (0.54; 2.68)	0.645	
3st Tertile		1.066 (0.53; 2.15)	0.858	0.944 (0.44; 2.03)	0.883	
G_Haemophilus	B		0.954*		0.955*	0.988
1st Tertile		Ref		Ref		
2st Tertile		0.932 (0.55; 1.57)	0.790	1.084 (0.61; 1.91)	0.781	
3st Tertile		1.000 (0.59; 1.69)	1.000	1.011 (0.57; 1.79)	0.971	
G_Cloacibacillus	B		0.607*		0.851*	0.988
1st Tertile		Ref		Ref		
2st Tertile		1.487 (0.68; 3.27)	0.324	1.244 (0.52; 2.97)	0.622	
3st Tertile		1.189 (0.53; 2.69)	0.678	1.255 (0.52; 3.03)	0.614	
G_Akkersmansia	B		0.545*		0.634*	0.988
1st Tertile		Ref		Ref		
2st Tertile		0.826 (0.48; 1.42)	0.491	0.953 (0.53; 1.72)	0.874	
3st Tertile		0.741 (0.43; 1.27)	0.275	0.765 (0.42; 1.38)	0.375	
		**Phylum**				
P_Actinobacteria	Q		0.205*		0.499*	0.626
1st tertile		Ref		Ref		
2st tertile		0.076 (-0.18; 0.33)	0.560	0.027 (-0.23; 0.29)	0.836	
3st tertile		0.228 (-0.03; 0.07)	0.081	0.146 (-0.11; 0.41)	0.268	
P_Bacteroidetes	Q		0.553*		0.539*	0.626
1st tertile		Ref		Ref		
2st tertile		0.007 (0.25; 0.26)	0.996	0.048 (-0.22; 0.31)	0.724	
3st tertile		-0.122 (-0.38; 0.13)	0.348	-0.099 (-0.36; 0.17)	0.462	
P_Firmicutes	Q		0.746*		0.626*	0.626
1st tertile		Ref		Ref		
2st tertile		-0.077 (-0.33; 0.18)	0.553	-0.088 (-0.35; 0.18)	0.515	
3st tertile		0.016 (-0.24; 0.27)	0.903	0.040 (-0.22; 0.30)	0.768	
P_Proteobacteria	Q		0.245*		0.208*	0.626
1st tertile		Ref		Ref		
2st tertile		0.024 (-0.23; 0.28)	0.852	0.014 (-0.29; 0.26)	0.919	
3st tertile		-0.175 (-0.43; 0.08)	0.177	-0.220 (-0.49; 0.05)	0.114	
**UPF Consumption 11 years**						
**Exposure:**	**Model**	**Crude model**		**Adjusted modela**		**FDR**
**UPF consumption**		**Beta/OR (IC95%)**	**P value**	**Beta/OR (IC95%)**	**P value**	**P value**
		**Genus**				
G_Bacteroides	Q		**0.002***		**0.016***	0.428
1st Tertile		Ref		Ref		
2st Tertile		-0.274 (-0.52; -0.03)	**0.030**	-0.254 (-0.50; -0.01)	**0.040**	
3st Tertile		-0.444 (-0.69; -0.20)	**<0.001**	-0.354 (-0.60; -0.11)	**0.005**	
G_Parabacteroides	Q		0.056*		0.263*	0.659
1st Tertile		Ref		Ref		
2st Tertile		-0.176 (-0.42; 0.07)	0.165	-0.155 (-0.41; 0.10)	0.226	
3st Tertile		-0.303 (-0.55; 0.05)	**0.017**	-0.205 (-0.46; 0.05)	0.117	
G_Prevotella	Q		**0.043***		0.482*	0.864
1st Tertile		Ref		Ref		
2st Tertile		0.236 (-0.01; 0.48)	0.063	0.138 (-0.11; 0.39)	0.276	
3st Tertile		0.303 (0.05; 0.55)	**0.017**	0.132 (-0.12; 0.39)	0.310	
G_Alistipes	Q		0.674*		0.778*	0.935
1st Tertile		Ref		Ref		
2st Tertile		-0.102 (-0.35; 0.15)	0.424	-0.041 (-0.29; 0.21)	0.750	
3st Tertile		-0.093 (-0.35; 0.16)	0.464	0.049 (-0.21; 0.31)	0.710	
G_Clostridium_Sensu_Stricto	Q		0.830*		0.712*	0.903
1st Tertile		Ref		Ref		
2st Tertile		0.019 (-0.23; 0.27)	0.881	-0.072 (-0.34; 0.20)	0.597	
3st Tertile		0.075 (-0.18; 0.32)	0.557	-0.114 (-0.39; 0.16)	0.415	
G_Anaerostipes	Q		0.966*		0.772*	0.935
1st Tertile		Ref		Ref		
2st Tertile		-0.032 (-0.287; 0.22)	0.800	0.028 (-0.22; 0.28)	0.826	
3st Tertile		-0.025 (-0.27; 0.23)	0.847	0.092 (-0.17; 0.35)	0.485	
G_Blautia	Q		0.490*		0.165*	0.592
1st Tertile		Ref		Ref		
2st Tertile		0.153 (-0.09; 0.40)	0.233	0.220 (-0.03; 0.48)	0.090	
3st Tertile		0.081 (-0.17; 0.33)	0.525	0.215 (-0.05; 0.48)	0.106	
G_Clostridium_xlva	Q		0.587*		0.689*	0.903
1st Tertile		Ref		Ref		
2st Tertile		-0.114 (-0.37; 0.14)	0.373	-0.119 (-0.38; 0.15)	0.394	

3st Tertile		-0.115 (-0.37; 0.14)	0.370	-0.079 (-0.36; 0.20)	0.577	
G_Coprococcus	Q		0.053*		0.106*	0.571
1st Tertile		Ref		Ref		
2st Tertile		0.294 (0.05; 0.54)	**0.021**	0.248 (-0.02; 0.52)	0.070	
3st Tertile		0.068 (-0.18; 0.32)	0.593	0.001 (-0.27; 0.28)	0.994	
G_Dorea	Q		0.057*		0.056*	0.571
1st Tertile		Ref		Ref		
2st Tertile		0.287 (0.04; 0.54)	**0.025**	0.335 (0.06; 0.61)	**0.017**	
3st Tertile		0.235 (-0.02; 0.48)	0.066	0.206 (-0.07; 0.49)	0.148	
G_Fusicatenibacter	Q		0.550*		0.592*	0.864
1st Tertile		Ref		Ref		
2st Tertile		0.085 (-0.17; 0.34)	0.505	0.110 (-0.15; 0.37)	0.403	
3st Tertile		-0.054 (-0.30; 0.20)	0.675	-0.009 (-0.27; 0.26)	0.947	
G_Roseburia	Q		**0.012***		**0.019***	0.428
1st Tertile		Ref		Ref		
2st Tertile		0.190 (-0.06; 0.44)	0.135	0.245 (-0.02; 0.51)	0.070	
3st Tertile		-0.189 (-0.44; 0.06)	0.136	-0.124 (-0.40; 0.15)	0.370	
G_Ruminococcus2	Q		0.736*		0.642*	0.903
1st Tertile		Ref		Ref		
2st Tertile		0.099 (-0.15; 0.35)	0.437	0.129 (-0.14; 0.40)	0.350	
3st Tertile		0.034 (-0.22; 0.29)	0.759	0.056 (-0.22; 0.33)	0.692	
G_Intestinibacter	Q		0.446*		0.497*	0.864
1st Tertile		Ref		Ref		
2st Tertile		-0.010 (-0.26; 0.24)	0.938	-0.002 (-0.27; 0.27)	0.990	
3st Tertile		0.135 (-0.11; 0.38)	0.289	0.139 (-0.13; 0.41)	0.316	
G_Romboutsia	Q		0.593*		0.712*	0.903
1st Tertile		Ref		Ref		
2st Tertile		-0.063 (-0.31; 0.19)	0.620	-0.072 (-0.33; 0.19)	0.590	
3st Tertile		0.067 (-0.18; 0.32)	0.597	0.034 (-0.24; 0.30)	0.803	
G_Clostridium_IV	Q		**0.046***		0.146*	0.571
1st Tertile		Ref		Ref		
2st Tertile		-0.243 (-0.49; 0.01)	0.056	-0.230 (-0.50; 0.04)	0.093	
3st Tertile		-0.298 (-0.55; -0.05)	**0.020**	-0.243 (-0.52; 0.03)	0.082	
G_Faecalibacterium	Q		0.355*		0.268*	0.659
1st Tertile		Ref		Ref		
2st Tertile		-0.122 (-0.37; 0.13)	0.342	-0.198 (-0.46;0.06)	0.132	
3st Tertile		-0.180 (-0.43; 0.07)	0.159	-0.174 (-0.44; 0.09)	0.193	
G_Gemmiger	Q		0.168*		0.584*	0.864
1st Tertile		Ref		Ref		
2st Tertile		-0.039 (-0.29; 0.21)	0.759	-0.009 (-0.27; 0.26)	0.947	
3st Tertile		-0.224 (-0.47; 0.02)	0.077	-0.126 (-0.39; 0.14)	0.360	
G_Oscillibacter	Q		0.766*		0.577*	0.864
1st Tertile		Ref		Ref		
2st Tertile		-0.087 (-0.33; 0.16)	0.498	-0.067 (-0.33; 0.20)	0.621	
3st Tertile		-0.073 (-0.32; 0.18)	0.568	0.073 (-0.20; 0.35)	0.596	
G_Ruminococcus	Q		0.132*		0.089*	0.571
1st Tertile		Ref		Ref		
2st Tertile		-0.217 (-0.47; 0.03)	0.088	-0.179 (-0.44; 0.09)	0.184	
3st Tertile		0.010 (-0.24; 0.26)	0.937	0.111 (-0.16; 0.38)	0.420	
G_Lachnospiracea_incertae_sedis	Q		0.934*		0.763*	0.935
1st Tertile		Ref		Ref		
2st Tertile		0.045 (-0.21; 0.30)	0.734	0.080 (-0.18; 0.34)	0.549	
3st Tertile		0.037 (-0.21; 0.29)	0.770	0.093 (-0.18; 0.36)	0.498	
G_Erysipelotrichaceae_incertae_sedis	B		0.119*		0.266*	0.659
1st Tertile		Ref		Ref		
2st Tertile		0.627 (0.37; 1.05)	0.076	0.623 (0.35; 1.11)	0.106	
3st Tertile		0.627 (0.37; 1.05)	0.076	0.749 (0.42; 1.34)	0.331	
G_Methanobrevibacter	B		560		0.306*	0.725
1st Tertile		Ref		Ref		
2st Tertile		1.134 (0.67; 1.90)	0.634	1.003 (0.56; 1.79)	0.993	
3st Tertile		1.309 (0.78; 2.21)	0.311	1.201 (0.66; 2.18)	0.548	
G_Methanosphaera	B		0.950*		0.916*	0.963
1st Tertile		Ref		Ref		
2st Tertile		1.062 (0.57; 1.97)	0.849	0.887 (0.43; 1.82)	0.743	
3st Tertile		0.960 (0.51; 1.80)	0.898	1.016 (0.49; 2.09)	0.966	
G_Actinomyces	B		**0.047***		0.081*	0.571
1st Tertile		Ref		Ref		
2st Tertile		0.803 (0.48; 1.33)	0.397	0.812 (0.47; 1.41)	0.462	
3st Tertile		1.513 (0.91; 2.51)	0.109	1.515 (0.86; 2.67)	0.150	
G_Rothia	B		0.258*		0.121*	0.571

1st Tertile		Ref		Ref		
2st Tertile		0.597 (0.31; 1.14)	0.116	0.567 (0.28; 1.16)	0.119	
3st Tertile		0.921 (0.51; 1.67)	0.788	1.445 (0.58; 2.25)	0.694	
G_Bifidobacterium	B		0.504*		0.811*	0.936
1st Tertile		Ref		Ref		
2st Tertile		1.143 (0.40; 3.26)	0.803	1.307 (0.37; 4.59)	0.676	
3st Tertile		2.053 (0.60; 7.01)	0.251	1.548 (0.39; 6.16)	0.535	
G_Collinsela	B		0.251		0.857*	0.941
1st Tertile		Ref		Ref		
2st Tertile		1.711 (0.61; 4.86)	0.314	1.294 (0.36; 4.59)	0.690	
3st Tertile		2.611 (0.80; 8.57)	0.113	1.444 (0.36; 5.80)	0.604	
G_Eggerthella	B		0.173*		0.523*	0.864
1st Tertile		Ref		Ref		
2st Tertile		0.806 (0.49; 1.33)	0.403	0.823 (0.46; 1.46)	0.507	
3st Tertile		0.615 (0.37; 1.02)	0.061	0.710 (0.39; 1.28)	0.256	
G_Gordonibacter	B		0.272*		0.805*	0.936
1st Tertile		Ref		Ref		
2st Tertile		0.729 (0.41; 1.28)	0.271	0.847 (0.45; 1.60)	0.609	
3st Tertile		0.635 (0.36; 1.13)	0.121	0.813 (0.42; 1.59)	0.545	
G_Olsenella	B		0.243*		0.549*	0.864
1st Tertile		Ref		Ref		
2st Tertile		0.798 (0.48; 1.33)	0.390	0.764 (0.42; 1.38)	0.370	
3st Tertile		1.239 (0.75; 2.05)	0.406	1.018 (0.57; 1.83)	0.953	
G_Senegalimassilia	B		**0.008***		0.162*	0.592
1st Tertile		Ref		Ref		
2st Tertile		1.238 (0.75; 2.05)	0.405	1.245 (0.68; 2.27)	0.475	
3st Tertile		2.217 (1.32; 3.72)	**0.003**	1.803 (0.98; 3.33)	0.060	
G_Slackia	B		0.274*		0.905*	0.941
1st Tertile		Ref		Ref		
2st Tertile		1.022 (0.61; 1.72)	0.934	0.900 (0.48; 1.67)	0.738	
3st Tertile		1.487 (0.87; 2.54)	0.148	1.027 (0.54; 1.94)	0.936	
G_Barnesiella	B		0.777*		0.968*	0.968
1st Tertile		Ref		Ref		
2st Tertile		1.210 (0.69; 2.12)	0.503	0.924 (0.50; 1.71)	0.802	
3st Tertile		1.161 (0.67; 2.02)	0.599	0.971 (0.52; 1.82)	0.928	
G_Butyricimonas	B		**0.020***		**0.010***	0.428
1st Tertile		Ref		Ref		
2st Tertile		1.804 (1.01; 2.23)	0.047	1.861 (0.98; 3.54)	0.058	
3st Tertile		2.237 (1.22; 4.09)	**0.009**	2.925 (1.44; 5.96)	**0.003**	
G_Coprobacter	B		0.134*		0.233*	0.659
1st Tertile		Ref		Ref		
2st Tertile		1.092 (0.50; 2.27)	0.825	1.022 (0.45; 2.34)	0.958	
3st Tertile		1.908 (0.94; 3.90)	0.076	1.802 (0.82; 3.98)	0.144	
G_Odoribacter	B		0.178*		0.171*	0.592
1st Tertile		Ref		Ref		
2st Tertile		1.613 (0.67; 3.88)	0.286	1.759 (0.66; 4.70)	0.260	
3st Tertile		2.485 (0.92; 6.70)	0.072	2.773 (0.94; 8.21)	0.065	
G_Porphyromonas	B		0.296*		0.194*	0.626
1st Tertile		Ref		Ref		
2st Tertile		0.775 (0.41; 1.47)	0.436	0.681 (0.34; 1.35)	0.269	
3st Tertile		1.272 (0.70; 2.31)	0.429	1.258 (0.66; 2.42)	0.490	
G_Paraprevotella	B		0.585*		0.961*	0.968
1st Tertile		Ref		Ref		
2st Tertile		0.887 (0.53; 1.47)	0.643	0.937 (0.53; 1.65)	0.821	
3st Tertile		1.159 (0.70; 1.92)	0.566	1.008 (0.57; 1.79)	0.977	
G_Lactobacillus	B		0.065*		0.332*	0.747
1st Tertile		Ref		Ref		
2st Tertile		1.090 (0.66; 1.81)	0.739	0.975 (0.55; 1.71)	0.929	
3st Tertile		1.803 (1.06; 3.05)	**0.028**	1.450 (0.80; 2.62)	0.217	
G_Lactococcus	B		0.973*		0.832*	0.938
1st Tertile		Ref		Ref		
2st Tertile		1.009 (0.48; 2.12)	0.980	1.140 (0.49; 2.67)	0.762	
3st Tertile		1.083 (0.52; 2.57)	0.832	1.309 (0.55; 3.14)	0.545	
G_Streptococcus	B		0.110*		0.137*	0.571
1st Tertile		Ref		Ref		
2st Tertile		2.394 (0.60; 9.49)	0.214	2.046 (0.42; 10.04)	0.378	
3st Tertile		0.609 (0.23; 1.63)	0.322	0.505 (0.15; 1.70)	0.271	
G_Christensenella	B		0.643*		0.779*	0.935
1st Tertile		Ref		Ref		
2st Tertile		1.164 (0.64; 2.12)	0.621	1.237 (0.63; 2.41)	0.532	

3st Tertile		0.867 (,.46; 1.62)	0.655	1.014 (0.50; 2.06)	0.968	
G_Anaerococcus	B		0.418*		0.393*	0.804
1st Tertile		Ref		Ref		
2st Tertile		0.695 (0.32; 1.49)	0.351	0.609 (0.27; 1.37)	0.232	
3st Tertile		1.144 (0.57; 2.29)	0.703	1.003 (0.46; 2.19)	0.993	
G_Ezakiella	B		0.725*		0.512*	0.834
1st Tertile		Ref		Ref		
2st Tertile		1.187 (0.54; 2.61)	0.671	1.153 (0.49; 2.70)	0.743	
3st Tertile		1.371 (0.63; 2.96)	0.423	1.606 (0.69; 3.72)	0.269	
G_Murdochiella	B		0.912*		0.707*	0.903
1st Tertile		Ref		Ref		
2st Tertile		1.138 (0.58; 2.24)	0.708	1.033 (0.48; 2.22)	0.934	
3st Tertile		1.138 (0.58; 2.24)	0.708	1.340 (0.62; 2.91)	0.459	
G_Parvimonas	B		0.609*		0.711*	0.903
1st Tertile		Ref		Ref		
2st Tertile		1.087(0.51; 2.31)	0.828	0.890 (0.39; 2.03)	0.782	
3st Tertile		1.412 (0.69; 2.91)	0.349	1.229 (0.55; 2.72)	0.611	
G_Mogibacterium	B		0.625*		0.669*	0.903
1st Tertile		Ref		Ref		
2st Tertile		0.966 (0.53; 1.75)	0.908	0.806 (0.41; 1.58)	0.528	
3st Tertile		1.253 (071; 2.23)	0.442	1.067 (0.56;2.05)	0.843	
G_Anaerofustis	B		0.625*		0.146*	0.571
1st Tertile		Ref		Ref		
2st Tertile		0.695 (0.32; 1.49)	0.351	0.648 (0.28; 1.52)	0.318	
3st Tertile		0.355 (0.14; 0.88)	**0.026**	0.359 (0.13; 1.01)	0.053	
G_Eubacterium	B		0.3713*		0.463*	0.850
1st Tertile		Ref		Ref		
2st Tertile		0.835 (0.43; 1.62)	0.594	0.809 (0.38; 1.72)	0.582	
3st Tertile		1.386 (0.67; 2.85)	0.376	1.330 (0.58; 3.04)	0.498	
G_Butyrivibrio	B		0.123*		0.123*	0.571
1st Tertile		Ref		Ref		
2st Tertile		1.091 (0.64; 1.86)	0.751	0.923 (0.50; 1.70)	0.798	
3st Tertile		1.658 (0.98; 2.80)	0.058	1.221 (0.67; 2.22)	0.514	
G_Clostridium_XLVb	B		0.256*		0.462*	0.850
1st Tertile		Ref		Ref		
2st Tertile		0.609 (0.23; 1.63)	0.322	0.544 (0.17; 1.75)	0.307	
3st Tertile		0.553 (0.21; 1.46)	0.230	0.385 (0.12; 1.20)	0.100	
G_Eisenbergiella	B		0.267*		0.230*	0.655
1st Tertile		Ref		Ref		
2st Tertile		0.661 (0.40; 1.10)	0.109	0.679 (0.38; 1.23)	0.199	
3st Tertile		0.863 (0.52; 1.43)	0.565	1.102 (0.61; 2.00)	0.750	
G_Howardella	B		**0.045***		0.056*	0.571
1st Tertile		Ref		Ref		
2st Tertile		1.993 (1.15; 3.45)	**0.014**	1.745 (0.92; 3.30)	0.087	
3st Tertile		1.609 (0.92; 2.80)	0.093	2.156 (1.14; 4.07)	**0.018**	
G_Peptococcus	B		0.241*		0.854*	0.938
1st Tertile		Ref		Ref		
2st Tertile		1.652 (0.91; 3.00)	0.100	1.204 (0.63; 2.31)	0.574	
3st Tertile		1.457 (0.80; 2.67)	0.222	1.115 (0.58; 2.16)	0.748	
G_Peptoniphilus	B		0.394*		0.179*	0.597
1st Tertile		Ref		Ref		
2st Tertile		0.657 (0.36; 1.20)	0.172	0.555 (0.28; 1.08)	0.083	
3st Tertile		0.839 (0.47; 1.50)	0.554	0.928 (0.49; 1.77)	0.822	
G_Clostridium_XI	B		0.864*		0.794*	0.936
1st Tertile		Ref		Ref		
2st Tertile		1.330 (0.68; 2.59)	0.400	0.837 (0.41; 1.72)	0.628	
3st Tertile		1.261 (0.65; 2.46)	0.497	0.737 (0.38; 1.62)	0.511	
G_Peptostreptococcus	B		**0.040***		**0.010***	0.428
1st Tertile		Ref		Ref		
2st Tertile		0.528 (0.28; 1.01)	0.054	0.704 (0.34; 1.45)	0.343	
3st Tertile		1.187 (0.67; 2.11)	0.558	1.919 (0.99; 3.72)	0.054	
G_Terrisporobacter	B		0.732*		0.941*	0.968
1st Tertile		Ref		Ref		
2st Tertile		0.833 (0.46; 1.51)	0.546	0.911 (0.48; 1.71)	0.771	
3st Tertile		1.044 (0.59; 1.86)	0.883	0.901 (0.47; 1.73)	0.754	
G_Anaerofilum	B		0.424*		0.682*	0.903
1st Tertile		Ref		Ref		
2st Tertile		0.815 (0.49; 1.36)	0.433	0.946 (0.54; 1.65)	0.844	
3st Tertile		1.145 (0.69; 1.91)	0.603	1.201 (0.68; 2.13)	0.531	
G_Anaerotruncus	B		0.474*		0.378*	0.795

1st Tertile		Ref		Ref		
2st Tertile		0.648 (0.32; 1.30)	0.222	0.756 (0.36; 1.59)	0.462	
3st Tertile		0.769 (0.38; 1.57)	0.470	1.267 (0.57; 2.82)	0.561	
G_Flavonifractor	B		0.077*		0.834*	0.938
1st Tertile		Ref		Ref		
2st Tertile		0.484 (0.25; 0.92)	**0.027**	0.812 (0.39; 1.69)	0.577	
3st Tertile		0.734 (0.38; 1.46)	0.391	0.826 (0.39; 1.73)	0.612	
G_Intestinimonas	B		**0.036***		0.640*	0.903
1st Tertile		Ref		Ref		
2st Tertile		0.587 (0.35; 0.99)	**0.047**	1.076 (0.60; 1.93)	0.805	
3st Tertile		0.523 (031; 0.89)	**0.017**	1.316 (0.73; 2.38)	0.366	
G_Pseudoflavonifractor	B		0.083*		**0.030***	0.428
1st Tertile		Ref		Ref		
2st Tertile		0.658 (0.36; 1.20)	0.172	0.547 (0.58; 1.09)	0.085	
3st Tertile		0.495 (0.26; 0.93)	**0.030**	0.371 (0.17; 0.80)	**0.011**	
G_Catenibacterium	B		0.643*		0.245*	0.659
1st Tertile		Ref		Ref		
2st Tertile		1.269 (0.76; 2.12)	0.362	0.941 (0.51; 1.75)	0.849	
3st Tertile		1.186 (0.71; 1.98)	0.514	1.511 (0.81; 2.81)	0.191	
G_Clostridium_XVIII	B		0.106*		0.587*	0.864
1st Tertile		Ref		Ref		
2st Tertile		0.494 (0.23; 1.05)	0.067	1.506 (0.68; 3.33)	0.311	
3st Tertile		0.468 (0.22; 0.99)	**0.047**	1.142 (0.54; 2.42)	0.729	
G_Coprobacillus	B		0.583*		0.060*	0.571
1st Tertile		Ref		Ref		
2st Tertile		1.186 (0.61; 2.30)	0.613	0.539 (0.24; 1.20)	0.132	
3st Tertile		0.824 (0.41; 1.67)	0.592	1.403 (0.68; 2.89)	0.358	
G_Holdemanella	B		**0.009***		0.103*	0.571
1st Tertile		Ref		Ref		
2st Tertile		1.155 (0.68; 1.95)	0.592	1.161 (0.63; 2.16)	0.635	
3st Tertile		2.342 (1.32;4.14)	**0.003**	2.009 (1.03; 2.91)	**0.040**	
G_Holdemania	B		0.555*		0.070*	0.571
1st Tertile		Ref		Ref		
2st Tertile		0.761 (0.46; 1.27)	0.297	0.604 (0.34; 1.06)	0.080	
3st Tertile		0.934 (0.56; 1.57)	0.795	1.123 (0.64; 1.99)	0.688	
G_Turicibacter	B		0.506*		0.369*	0.795
1st Tertile		Ref		Ref		
2st Tertile		0.759 (0.37; 1.58)	0.459	1.250 (0.58; 2.69)	0.567	
3st Tertile		1.176 (0.53; 2.59)	0.687	1.858 (0.79; 4.39)	0.159	
G_Acidaminococcus	B		0.978*		0.271*	0.659
1st Tertile		Ref		Ref		
2st Tertile		1.069 (0.52; 2.19)	0.855	0.543 (0.25; 1.19)	0.127	
3st Tertile		1.000 (0.48; 2.07)	1.000	0.633 (0.29; 1.37)	0.245	
G_Phascolarctobacterium	B		0.140*		0.116*	0.571
1st Tertile		Ref		Ref		
2st Tertile		1.051 (0.57; 1.95)	0.874	0.711 (0.37; 1.36)	0.301	
3st Tertile		0.867 (0.47; 1.59)	0.644	1.476 (0.71; 3.06)	0.295	
G_Allisonella	B		**0.002***		0.063*	0.571
1st Tertile		Ref		Ref		
2st Tertile		2.490 (1.45; 4.27)	**0.001**	0.931 (0.51; 1.71)	0.819	
3st Tertile		2.029 (1.18; 3.49)	**0.010**	1.769 (0.97; 3.23)	0.063	
G_Dialister	B		0.503*		0.448*	0.850
1st Tertile		Ref		Ref		
2st Tertile		1.036 (0.61; 1.75)	0.894	1.249 (0.71; 2.20)	0.441	
3st Tertile		1.341 (0.79; 2.28)	0.280	1.453 (0.81; 2.61)	0.209	
G_Megamonas	B		**0.038***		0.138*	0.571
1st Tertile		Ref		Ref		
2st Tertile		1.437 (0.76; 2.71)	0.263	1.690 (0.86; 3.30)	0.125	
3st Tertile		2.194 (1.19; 4.03)	**0.012**	1.932 (0.99; 3.77)	0.054	
G_Megasphaera	B		0.883*		0.555*	0.864
1st Tertile		Ref		Ref		
2st Tertile		1.110 (0.59; 2.09)	0.746	1.389 (0.65; 2.95)	0.392	
3st Tertile		0.947 (0.49; 1.81)	0.868	1.486 (0.71; 3.12)	0.296	
G_Mitsuokella	B		0.892*		0.136*	0.571
1st Tertile		Ref		Ref		
2st Tertile		1.157 (0.63; 2.13)	0.640	1.879 (0.88; 3.99)	0.101	
3st Tertile		1.051 (0.57; 1.95)	0.874	2.060 (0.98; 4.32)	0.056	
G_Veillonella	B		0.463*		0.502*	0.864
1st Tertile		Ref		Ref		
2st Tertile		1.043 (0.59; 1.85)	0.884	1.200 (0.65; 2.21)	0.560	

3st Tertile		0.757 (0.44; 1.31)	0.325	1.459 (0.78; 2.74)	0.214	
G_Fusobacterium	B		0.489*		0.920*	0.963
1st Tertile		Ref		Ref		
2st Tertile		1.067 (0.53; 2.15)	0.858	1.177 (0.53; 2.60)	0.686	
3st Tertile		0.687 (0.32; 1.48)	0.337	1.072 (0.48; 2.41)	0.865	
G_Victivallis	B		0.952*		0.462*	0.850
1st Tertile		Ref		Ref		
2st Tertile		0.929 (0.54; 1.58)	0.786	1.243 (0.70; 2.21)	0.458	
3st Tertile		0.929 (0.54; 1.58)	0.786	1.454 (0.80; 2.64)	0.218	
G_Parasutterella	B		0.771*		0.206*	0.639
1st Tertile		Ref		Ref		
2st Tertile		0.815 (0.46; 1.43)	0.475	0.604 (0.32; 1.14)	0.121	
3st Tertile		0.919 (0.52; 1.62)	0.722	0.592 (0.31; 1.12)	0.108	
G_Sutterella	B		0.552*		0.595*	0.864
1st Tertile		Ref		Ref		
2st Tertile		1.000 (0.59; 1.71)	1.000	1.084 (0.60; 1.96)	0.789	
3st Tertile		0.777 (0.46; 1.32)	0.348	0.808 0.44; 1.47)	0.484	
G_Bilophila	B		0.842*		0.574*	0.864
1st Tertile		Ref		Ref		
2st Tertile		0.800 (0.37; 1.71)	0.564	0.939 (0.42; 2.12)	0.881	
3st Tertile		0.858 (040; 1.85)	0.696	1.447 (0.60; 3.48)	0.410	
G_Desulfovibrio	B		0.895*		0.962*	0.968
1st Tertile		Ref		Ref		
2st Tertile		0.867 (0.47; 1,59)	0.644	1.099 (0.56; 2.16)	0.782	
3st Tertile		0.952 (0.52; 1.76)	0.876	1.056 (0.53; 2.09)	0.876	
G_Campylobacter	B		0.553*		0.108*	0.571
1st Tertile		Ref		Ref		
2st Tertile		1.101 (0.60; 2.02)	0.757	0.863 (0.45; 1.67)	0.661	
3st Tertile		1.374 (0.80; 2.48)	0.294	1.633 (0.87; 3.08)	0.130	
G_Succinivibrio	B		0.464*		0.380*	0.795
1st Tertile		Ref		Ref		
2st Tertile		1.694 (0.73; 3.90)	0.216	0.705 (0.27; 1.86)	0.479	
3st Tertile		1.337 (0.56; 3.18)	0.511	1.330 (0.55; 3.23)	0.527	
G_Escherichia/Shigella	B		0.747*		0.326*	0.747
1st Tertile		Ref		Ref		
2st Tertile		1.318 (0.63; 2.74)	0.459	0.535 (0.24; 1.22)	0.136	
3st Tertile		1.066 (0.53; 2.15)	0.858	0.643 (0.27; 1.52)	0.316	
G_Haemophilus	B		0.954*		0.456*	0.850
1st Tertile		Ref		Ref		
2st Tertile		0.932 (0.55; 1.57)	0.790	0.884 (0.50; 1.58)	0.678	
3st Tertile		1.000 (0.59; 1.69)	1.000	0.693 (0.38; 1.25)	0.221	
G_Cloacibacillus	B		0.607*		0.213*	0.639
1st Tertile		Ref		Ref		
2st Tertile		1.487 (0.68; 3.27)	0.324	0.703 (0.27; 1.82)	0.467	
3st Tertile		1.189 (0.53; 2.69)	0.678	1.538 (0.66; 3.59)	0.320	
G_Akkersmansia	B		0.545*		0.894*	0.941
1st Tertile		Ref		Ref		
2st Tertile		0.827 (0.48; 1.42)	0.491	0.904 (0.50; 1.63)	0.736	
3st Tertile		0.741 (0.43; 1.27)	0.275	0.867 (0.47; 1.59)	0.645	
		**Phylum**				
P_Actinobacteria	Q		**0.006***		**0.032***	0.090
1st tertile		Ref		Ref		
2st tertile		0.041 (-0.21; 0.29)	0.747	0.049 (-0.21; 0.31)	0.709	
3st tertile		0.369 (0.12; 0.62)	**0.004**	0.325 (0.60; 0.59)	**0.016**	
P_Bacteroidetes	Q		0.900*		0.436*	0.550
1st tertile		Ref		Ref		
2st tertile		0.005 (-0.25; 0.26)	0.966	-0.078 (-0.45; 0.09)	0.565	
3st tertile		-0.048 (-0.30; 0.20)	0.708	-0.178 (-0.45; 0.09)	0.199	
P_Firmicutes	Q		0.926*		0.550*	0.550
1st tertile		Ref		Ref		
2st tertile		0.024 (-0.23; 0.28)	0.854	0.126 (-0.14; 0.39)	0.353	
3st tertile		-0.027 (-0.28; 0.23)	0.836	0.135 (-0.14; 0.41)	0.329	
P_Proteobacteria	Q		0.052*		**0.045***	0.090
1st tertile		Ref		Ref		
2st tertile		-0.308 (-0.56; -0.06)	0.016	-0.344 (-0.62; -0.07)	**0.014**	
3st tertile		-0.121 (-0.37; 0.13)	0.343	-0.139 (-0.42; 0.14)	0.327	
**UPF Consumption 12 years**						
**Exposure:**	**Model**	**Crude model**		**Adjusted model#**		**FDR**
**UPF consumption**		**Beta/OR (IC95%)**	**P value**	**Beta/OR (IC95%)**	**P value**	**P value**
**Genus**						

G_Bacteroides	Q		**0.005***		0.094*	0.495
1st Tertile		Ref		Ref		
2st Tertile		0.023 (-0.22; 0.27)	0.853	0.112 (-0.13; 0.35)	0.363	
3st Tertile		-0.349 (-0.60; -0.11)	0.006	-0.156 (-0.40; 0.09)	0.211	
G_Parabacteroides	Q		0.070*		0.449*	0.768
1st Tertile		Ref		Ref		
2st Tertile		-0.005 (-0.25; 0.24)	0.966	0.033 (-0.22; 0.28)	0.797	
3st Tertile		-0.257 (-0.51; -0.01)	0.043	-0.121 (-0.37; 0.13)	0.347	
G_Prevotella	Q		**0.019***		0.477*	0.775
1st Tertile		Ref		Ref		
2st Tertile		0.078 (-0.17; 0.33)	0.538	0.005 (-0.24; 0.25)	0.977	
3st Tertile		0.342 (0.09; 0.59)	0.007	0.136 (-0.11; 0.39)	0.287	
G_Alistipes	Q		0.326*		0.818*	0.932
1st Tertile		Ref		Ref		
2st Tertile		0.017 (-0.23; 0.27)	0.892	0.066 (-0.19; 0.32)	0.607	
3st Tertile		-0.156 (-0.40; 0.09)	0.220	-0.008 (-0.26; 0.25)	0.953	
G_Clostridium_Sensu_Stricto	Q		0.067*		0.442*	0.768
1st Tertile		Ref		Ref		
2st Tertile		0.165 (-0.08; 0.42)	0.194	0.082 (-0.19; 0.35)	0.548	
3st Tertile		0.296 (0.05; 0.54)	**0.021**	0.175 (-0.09; 0.45)	0.202	
G_Anaerostipes	Q		0.480*		0.714*	0.892
1st Tertile		Ref		Ref		
2st Tertile		-0.144 (-0.40; 0.11)	0.261	-0.096 (-0.35; 0.16)	0.455	
3st Tertile		-0.122 (-0.37; 0.13)	0.341	-0.011 (-0.26; 0.24)	0.932	
G_Blautia	Q		0.302*		0.497*	0.776
1st Tertile		Ref		Ref		
2st Tertile		0.103 (-0.15; 0.35)	0.420	0.152 (-0.10; 0.41)	0.241	
3st Tertile		-0.095 (-0.34; 0.16)	0.457	0.062 (-0.20; 0.32)	0.637	
G_Clostridium_xlva	Q		0.256*		0.320*	0.695
1st Tertile		Ref		Ref		
2st Tertile		-0.044 (-0.29; 0.21)	0.731	-0.083 (-0.35; 0.19)	0.549	
3st Tertile		-0.201 (-0.45; 0.05)	0.116	-0.209 (-0.48; 0.06)	0.134	
G_Coprococcus	Q		0.637*		0.634*	0.863
1st Tertile		Ref		Ref		
2st Tertile		-0.062 (-0.31; 0.19)	0.628	-0.109 (0.38; 0.16)	0.422	
3st Tertile		-0.121 (-0.37; 0.13)	0.343	-0.117 (-.39; 0.15)	0.396	
G_Dorea	Q		0.860*		0.740*	0.892
1st Tertile		Ref		Ref		
2st Tertile		0.062 (-0.19; 0.31)	0.627	0.106 (-0.17; 0.38)	0.451	
3st Tertile		0.058 (-0.20; 0.31)	0.649	0.076 (-0.20; 0.35)	0.591	
G_Fusicatenibacter	Q		0.511*		0.752*	0.892
1st Tertile		Ref		Ref		
2st Tertile		-0.133 (-0.38; 0.12)	0.299	-0.081 (-0.34; 0.18)	0.536	
3st Tertile		-0.123 (-0.37; 0.13)	0.336	0.008 (-0.25; 0.27)	0.952	
G_Roseburia	Q		0.104*		0.406*	0.768
1st Tertile		Ref		Ref		
2st Tertile		-0.132 (-0.38; 0.12)	0.302	-0.094 (-0.36; 0.17)	0.488	
3st Tertile		-0.272 (-0.52; -0.02)	**0.034**	-0.184 (-0.45; 0.09)	0.180	
G_Ruminococcus2	Q		0.142*		**0.047***	0.495
1st Tertile		Ref		Ref		
2st Tertile		0.123 (-0.13; 0.37)	0.337	0.167 (-0.10; 0.43)	0.222	
3st Tertile		0.252 (0.02; 0.50)	**0.048**	0.341 (0.07; 0.61)	0.013	
G_Intestinibacter	Q		0.559*		0.395*	0.768
1st Tertile		Ref		Ref		
2st Tertile		-0.016 (-0.27; 0.23)	0.899	-0.096 (-0.36; 0.17)	0.478	
3st Tertile		0.110 (-0.14; 0.36)	0.386	0.088 (-0.18; 0.36)	0.518	
G_Romboutsia	Q		0.735*		0.787*	0.910
1st Tertile		Ref		Ref		
2st Tertile		0.055 (-0.20; 0.31)	0.667	0.053 (-0.21; 0.32)	0.692	
3st Tertile		0.010 (-0.15; 0.35)	0.434	0.093 (-0.17; 0.36)	0.491	
G_Clostridium_IV	Q		0.332*		0.250*	0.654
1st Tertile		Ref		Ref		
2st Tertile		0.079 (-0.17; 0.33)	0.532	0.083 (-0.18; 0.35)	0.542	
3st Tertile		-0.110 (-0.36; 0.14)	0.391	-0.142 (-0.41; 0.13)	0.302	
G_Faecalibacterium	Q		**0.037***		0.164*	0.561
1st Tertile		Ref		Ref		
2st Tertile		-0.040 (-0.29; 0.21)	0.754	-0.091 (-0.35; 0.17)	0.487	
3st Tertile		-0.302 (-0.55; -0.05)	**0.018**	-0.248 (-0.51; 0.01)	0.061	
G_Gemmiger	Q		0.158*		0.617*	0.863
1st Tertile		Ref		Ref		

2st Tertile		-0.102 (-0.35; 0.15)	0.423	-0.058 (-0.32; 0.20)	0.662	
3st Tertile		-0.243 (-0.49; 0.01)	0.056	-0.132 (-0.40; 0.13)	0.327	
G_Oscillibacter	Q		0.381*		0.995*	0.995
1st Tertile		Ref		Ref		
2st Tertile		-0.106 (-0.36; 0.15)	0.408	-0.013 (-0.28; 0.25)	0.925	
3st Tertile		-0.176 (-0.43; 0.07)	0.168	-0.003 (-0.27; 0.26)	0.980	
G_Ruminococcus	Q		0.204*		0.113*	0.495
1st Tertile		Ref		Ref		
2st Tertile		-0.227 (-0.48; 0.02)	0.075	-0.278 (-0.54; -0.15)	0.038	
3st Tertile		-0.118 (-0.37; 0.13)	0.356	-0.113 (-0.38; 0.15)	0.405	
G_Lachnospiracea_incertae_sedis	Q		0.950*		0.776*	0.909
1st Tertile		Ref		Ref		
2st Tertile		0.030 (-0.22; 0.28)	0.816	0.080 (-0.18; 0.34)	0.547	
3st Tertile		-0.010 (-0.26; 0.24)	0.939	0.085 (-0.18; 0.35)	0.529	
G_Erysipelotrichaceae_incertae_sedis	B		**0.039***		0.122*	0.495
1st Tertile		Ref		Ref		
2st Tertile		1.241 (0.77; 2.07)	0.405	1.480 (0.84; 2.61)	0.176	
3st Tertile		0.631 (0.37; 1.07)	0.090	0.814 (0.54; 1.47)	0.496	
G_Methanobrevibacter	B		**0.006***		**0.009***	0.495
1st Tertile		Ref		Ref		
2st Tertile		1.896 (1.13; 3.19)	0.016	2.256 (1.25; 4.07)	0.007	
3st Tertile		2.211 (1.31; 3.75)	0.003	2.149 (1.18; 3.89)	0.012	
G_Methanosphaera	B		0.127*		0.081*	0.495
1st Tertile		Ref		Ref		
2st Tertile		0.513 (0.27; 0.98)	0.043	0.432 (0.21; 0.91)	0.026	
3st Tertile		0.803 (0.44; 1.46)	0.471	0.649 (0.33; 1.28)	0.214	
G_Actinomyces	B		0.512*		0.281*	0.695
1st Tertile		Ref		Ref		
2st Tertile		0.861 (0.52; 1.43)	0.560	0.748 (0.43; 1.30)	0.306	
3st Tertile		1.160 (0.70; 1.92)	0.564	1.166 (0.67; 2.03)	0.587	
G_Rothia	B		0.617*		0.669*	0.881
1st Tertile		Ref		Ref		
2st Tertile		1.369 (0.73; 2.56)	0.326	1.331 (0.67; 2.65)	0.416	
3st Tertile		1.184 (0.63; 2.24)	0.604	1.314 (0.65; 2.66)	0.447	
G_Bifidobacterium	B		0.504*		0.720*	0.892
1st Tertile		Ref		Ref		
2st Tertile		2.053 (0.60; 7.01)	0.251	1.452 (0.38; 5.51)	0.583	
3st Tertile		1.143 (0.40; 3.26)	0.803	0.812 (0.23; 2.93)	0.750	
G_Collinsela	B		0.117*		0.313*	0.695
1st Tertile		Ref		Ref		
2st Tertile		0.991 (0.38; 2.59)	0.986	0.806 (0.25; 2.57)	0.715	
3st Tertile		4.739 (1.00; 22.41)	0.050	2.887 (0.54; 15.46)	0.216	
G_Eggerthella	B		**0.015***		0.084*	0.495
1st Tertile		Ref		Ref		
2st Tertile		0.707 (0.43; 1.17)	0.178	0.847 (0.48; 1.50)	0.571	
3st Tertile		0.468 (0.28; 0.78)	0.004	0.523 (0.29; 0.94)	0.031	
G_Gordonibacter	B		0.069*		0.320*	0.695
1st Tertile		Ref		Ref		
2st Tertile		0.798 (0.46; 1.39)	0.423	0.749 (0.40; 1.41)	0.370	
3st Tertile		0.499 (0.28; 0.90)	0.022	0.602 (0.31; 1.18)	0.138	
G_Olsenella	B		0.150*		0.477*	0.775
1st Tertile		Ref		Ref		
2st Tertile		1.124 (0.67; 1.88)	0.656	0.843 (0.47; 1.52)	0.568	
3st Tertile		1.622 (0.97; 2.70)	0.063	1.203 (0.68; 2.14)	0.529	
G_Senegalimassilia	B		**0.002***		**0.032***	0.495
1st Tertile		Ref		Ref		
2st Tertile		1.159 (0.70; 1.92)	0.565	1.037 (0.57; 1.88)	0.905	
3st Tertile		2.387 (1.42; 4.01)	**0.001**	2.079 (1.12; 3.85)	**0.020**	
G_Slackia	B		**0.021***		0.194*	0.620
1st Tertile		Ref		Ref		
2st Tertile		1.499 (0.89; 2.52)	0.127	1.495 (0.81; 2.76)	0.198	
3st Tertile		2.136 (1.25; 3.67)	**0.006**	1.748 (0.93; 3.29)	0.084	
G_Barnesiella	B		0.124*		0.237*	0.639
1st Tertile		Ref		Ref		
2st Tertile		0.848 (0.49; 1.46)	0.551	0.928 (0.51; 1.68)	0.806	
3st Tertile		1.526 (0.85; 2.74)	0.153	1.552 (0.82; 2.94)	0.178	
G_Butyricimonas	B		0.082*		0.139*	0.515
1st Tertile		Ref		Ref		
2st Tertile		1.672 (0.93; 3.00)	0.085	1.694 (0.89; 3.24)	0.111	
3st Tertile		1.855 (1.02; 3.37)	**0.042**	1.836 (0.93; 3.61)	0.078	

G_Coprobacter	B		0.190*		0.114*	0.495
1st Tertile		Ref		Ref		
2st Tertile		1.450 (0.73; 2.87)	0.288	1.556 (0.74; 3.29)	0.247	
3st Tertile		0.734 (0.34; 1.61)	0.451	0.654 (0.28; 1.54)	0.331	
G_Odoribacter	B		0.122*		0.109*	0.495
1st Tertile		Ref		Ref		
2st Tertile		3.007 (1.05; 8.63)	0.041	3.601 (1.09; 11.90)	0.036	
3st Tertile		1.439 (0.61; 3.38)	0.403	1.524 (0.58; 3.96)	0.389	
G_Porphyromonas	B		0.821*		0.871*	0.957
1st Tertile		Ref		Ref		
2st Tertile		1.112 (0.61; 2.04)	0.732	1.129 (0.59; 2.16)	0.716	
3st Tertile		0.914 (0.49; 1.70)	0.776	0.951 (049; 1.85)	0.883	
G_Paraprevotella	B		0.078*		0.370*	0.766
1st Tertile		Ref		Ref		
2st Tertile		1.426 (0.85; 2.38)	0.175	1.315 (0.74; 2.32)	0.344	
3st Tertile		1.798 (1.08; 3.00)	**0.025**	1.494 (0.85; 2.64)	0.166	
G_Lactobacillus	B		0.061*		0.050*	0.495
1st Tertile		Ref		Ref		
2st Tertile		0.753 (0.45; 1.25)	0.275	0.610 (0.34; 1.06)	0.080	
3st Tertile		1.417 (0.83; 2.40)	0.275	1.198 (0.66; 2.16	0.550	
G_Lactococcus	B		0.837*		0.588*	0.858
1st Tertile		Ref		Ref		
2st Tertile		0.753 (0.45; 1.25)	0.275	1.548 (0.66; 3.61)	0.311	
3st Tertile		1.417 (0.84; 2.40)	0.196	1.364 (0.57; 3.25)	0.484	
G_Streptococcus	B		0.365*		0.741*	0.892
1st Tertile		Ref		Ref		
2st Tertile		2.053 (0.60; 7.01)	0.251	1.700 (0.40; 7.18)	0.470	
3st Tertile		0.873 (0.33; 2.34)	0.788	1.016 (0.32; 3.20)	0.979	
G_Christensenella	B		0.378*		0.426*	0.768
1st Tertile		Ref		Ref		
2st Tertile		1.516 (0.81; 2.84)	0.195	1.415 (0.71; 2.83)	0.325	
3st Tertile		1.448 (0.77; 2.84)	0.251	1.566 (0.78; 3.15)	0.208	
G_Anaerococcus	B		0.760*		0.545*	0.808
1st Tertile		Ref		Ref		
2st Tertile		0.874 (0.41; 1.84)	0.723	0.801 (0.35; 1.81)	0.595	
3st Tertile		1.151 (0.57; 2.34)	0.698	1.254 (0.58; 2.74)	0.569	
G_Ezakiella	B		**0.018***		**0.020***	0.495
1st Tertile		Ref		Ref		
2st Tertile		0.563 (0.27; 1.16)	0.117	0.521 (0.24; 1.15)	0.108	
3st Tertile		0.305 (0.13; 0.71)	0.006	0.295 (0.12; 0.72)	0.007	
G_Murdochiella	B		0.565*		0.934*	0.967
1st Tertile		Ref		Ref		
2st Tertile		0.760 (0.39; 1.48)	0.418	0.873 (0.42; 1.83)	0.720	
3st Tertile		0.713 (0.36; 1.40)	0.324	0.912 (0.43; 1.92)	0.809	
G_Parvimonas	B		0.639*		0.510*	0.783
1st Tertile		Ref		Ref		
2st Tertile		0.722 (0.35; 1.49)	0.376	0.696 (0.31; 1.55)	0.375	
3st Tertile		0.777 (0.38; 1.58)	0.487	0.656 (0.30; 1.43)	0.288	
G_Mogibacterium	B		**0.023***		0.203*	0.620
1st Tertile		Ref		Ref		
2st Tertile		1.010 (0.54; 1.88)	0.974	0.975 (0.49; 1.95)	0.943	
3st Tertile		1.988 (1.11; 3.55)	**0.020**	1.612 (0.84; 3.07)	0.146	
G_Anaerofustis	B		0.145*		0.155*	0.552
1st Tertile		Ref		Ref		
2st Tertile		1.763 (0.80; 3.91)	0.163	2.087 (0.87; 5.02)	0.100	
3st Tertile		0.811 (0.32; 2.03)	0.655	0.993 (0.36; 2.77)	0.989	
G_Eubacterium	B		0.169*		0.280*	0.695
1st Tertile		Ref		Ref		
2st Tertile		1.384 (0.71; 2.67)	0.334	1.318 (0.63; 2.75)	0.462	
3st Tertile		1.970 (0.97; 4.01)	0.061	1.949 (0.86; 4.43)	0.111	
G_Butyrivibrio	B		0.381*		0.964*	0.975
1st Tertile		Ref		Ref		
2st Tertile		1.260 (0.74; 2.14)	0.392	1.079 (0.59; 1.96)	0.805	
3st Tertile		1.448 (0.86; 2.45)	0.167	1.069 (0.59; 1.94)	0.826	
G_Clostridium_XLVb	B		0.896*		0.602*	0.863
1st Tertile		Ref		Ref		
2st Tertile		1.111 (0.43; 2.84)	0.826	1.140 (0.39; 3.34)	0.812	
3st Tertile		0.893 (0.36; 2.19)	0.804	0.694 (0.26; 1.86)	0.468	
G_Eisenbergiella	B		0.741*		0.911*	0.967
1st Tertile		Ref		Ref		

2st Tertile		1.122 (0.68; 1.86)	0.654	1.1233 (0.63; 2.02)	0.672	
3st Tertile		0.920 (0.56; 1.52)	0.745	1.043 (0.58; 1.88)	0.888	
G_Howardella	B		0.057*		0.128*	0.495
1st Tertile		Ref		Ref		
2st Tertile		0.868 (0.50; 1.49)	0.610	0.715 (0.31; 1.34)	0.297	
3st Tertile		1.598 (0.95; 2.69)	0.078	1.339 (0.74; 2.43)	0.337	
G_Peptococcus	B		0.084*		0.532*	0.803
1st Tertile		Ref		Ref		
2st Tertile		0.919 (0.50; 1.68)	0.784	0.819 (0.42; 1.59)	0.558	
3st Tertile		1.658 (0.94; 2.92)	0.081	1.182 (0.63; 2.21)	0.602	
G_Peptoniphilus	B		0.561*		0.436*	0.768
1st Tertile		Ref		Ref		
2st Tertile		1.103 (0.62; 1.96)	0.740	1.200 (0.63; 2.27)	0.576	
3st Tertile		0.799 (0.44; 1.46)	0.465	.0772 90.39; 1.51)	0.450	
G_Clostridium_XI	B		0.253*		0.128*	0.495
1st Tertile		Ref		Ref		
2st Tertile		0.743 (0.38; 1.48)	0.399	0.752 (0.35; 1.62)	0.466	
3st Tertile		1.306 (0.70; 2.45)	0.406	1.555 (0.77; 3.12)	0.215	
G_Peptostreptococcus	B		0.315*		0.105*	0.495
1st Tertile		Ref		Ref		
2st Tertile		0.753 (0.41; 1.39)	0.366	0.571 (0.28; 1.15)	0.116	
3st Tertile		1.203 (0.67; 2.14)	0.531	1.164 (0.61; 2.22)	0.645	
G_Terrisporobacter	B		0.337*		0.446*	0.768
1st Tertile		Ref		Ref		
2st Tertile		0.654 (0.37; 1.17)	0.153	0.671 (0.36; 1.26)	0.217	
3st Tertile		0.753 (0.43; 1.33)	0.330	0.902 (0.48; 1.69)	0.749	
G_Anaerofilum	B		0.193*		0.095*	0.495
1st Tertile		Ref		Ref		
2st Tertile		1.511 (0.91; 2.51)	0.110	1.725 (0.98; 3.02)	0.057	
3st Tertile		1.015 (0.61; 1.68)	0.955	1.015 (0.57; 1.79)	0.960	
G_Anaerotruncus	B		0.977*		0.924*	0.967
1st Tertile		Ref		Ref		
2st Tertile		0.934 (0.48; 1.83)	0.841	1.094 (0.52; 2.32)	0.814	
3st Tertile		0.990 (0.50; 1.95)	0.977	1.163 (0.55; 2.46)	0.693	
G_Flavonifractor	B		0.710*		0.209*	0.620
1st Tertile		Ref		Ref		
2st Tertile		0.943 (0.51; 1.73)	0.850	0.965 (0.48; 1.94)	0.920	
3st Tertile		1.219 (0.65; 2.30)	0.540	1.753 (0.84; 3.66)	0.135	
G_Intestinimonas	B		0.328*		0.683*	0.881
1st Tertile		Ref		Ref		
2st Tertile		0.742 (0.44; 1.24)	0.258	0.774 (0.43; 1.38)	0.383	
3st Tertile		0.690 (0.41; 1.16)	0.162	0.867 (0.49; 1.55)	0.631	
G_Pseudoflavonifractor	B		0.136*		0.747*	0.892
1st Tertile		Ref		Ref		
2st Tertile		0.770 (0.43; 1.39)	0.387	0.909 (0.46; 1.81)	0.786	
3st Tertile		0.524 (0.28; 0.99)	**0.046**	0.751 (0.36; 1.57)	0.448	
G_Catenibacterium	B		**0.047***		0.492*	0.776
1st Tertile		Ref		Ref		
2st Tertile		0.981 (0.59; 1.63)	0.942	0.947 (0.51; 1.75)	0.861	
3st Tertile		1.728 (1.04; 2.87)	**0.035**	1.332 (0.72; 2.46)	0.360	
G_Clostridium_XVIII	B		0.664*		0.314*	0.695
1st Tertile		Ref		Ref		
2st Tertile		0.936 (0.48; 1.81)	0.843	0.853 (0.40; 1.81)	0.678	
3st Tertile		1.272 (0.63; 2.55)	0.498	1.549 (0.71; 3.40)	0.275	
G_Coprobacillus	B		0.085*		0.065*	0.495
1st Tertile		Ref		Ref		
2st Tertile		0.467 (0.23; 0.96)	0.039	0.438 (0.20; 0.98)	0.045	
3st Tertile		0.960 (0.51; 1.80)	0.989	1.080 (0.54; 2.17)	0.829	
G_Holdemanella	B		**0.0008***		**0.036***	0.495
1st Tertile		Ref		Ref		
2st Tertile		1.449 (0.86; 2.44)	0.162	1.153 (0.62; 2.14)	0.648	
3st Tertile		3.002 (1.69; 5.33)	<0.001	2.329 (1.20; 4.55)	**0.013**	
G_Holdemania	B		0.834*		0.348*	0.737
1st Tertile		Ref		Ref		
2st Tertile		1.122 (0.68; 1.86)	0.656	1.134 (0.65; 1.98)	0.659	
3st Tertile		1.159 (0.70; 1.92)	0.566	1.503 (0.86; 2.64)	0.157	
G_Turicibacter	B		0.234*		0.682*	0.881
1st Tertile		Ref		Ref		
2st Tertile		0.990 (0.50; 1.98)	0.978	0.884 (0.41; 1.89)	0.752	
3st Tertile		1.844 (0.84; 4.06)	0.129	1.267 (0.55; 2.98)	0.556	

G_Acidaminococcus	B		0.946*		0.925*	0.967
1st Tertile		Ref		Ref		
2st Tertile		0.886 (0.44; 1.80)	0.738	1.164 (0.54; 2.52)	0.700	
3st Tertile		0.947 (0.47; 1.91)	0.880	1.115 (0.51; 2.46)	0.788	
G_Phascolarctobacterium	B		0.115*		0.105*	0.495
1st Tertile		Ref		Ref		
2st Tertile		1.807 (0.89; 2.89)	0.113	1.528 (0.80; 2.94)	0.203	
3st Tertile		1.783 (0.98; 3.24)	0.058	2.092 (1.05; 4.18)	**0.037**	
G_Allisonella	B		0.322*		0.637*	0.863
1st Tertile		Ref		Ref		
2st Tertile		1.292 (0.77; 2.16)	0.332	1.317 (0.72; 2.39)	0.367	
3st Tertile		1.478 (0.88; 2.47)	0.137	1.249 (0.69; 2.25)	0.467	
G_Dialister	B		0.326*		0.479*	0.775
1st Tertile		Ref		Ref		
2st Tertile		0.695 (0.41; 1.17)	0.171	0.704 (0.40; 1.24)	0.225	
3st Tertile		0.952 (0.56; 1.62)	0.856	0.840 (0.47; 1.50)	0.557	
G_Megamonas	B		**0.037***		0.107*	0.495
1st Tertile		Ref		Ref		
2st Tertile		1.729 (0.93; 3.21)	0.083	1.580 (0.81; 3.10)	0.182	
3st Tertile		2.202 (1.20; 4.04	**0.011**	2.043 (1.05; 3.97)	0.035	
G_Megasphaera	B		**0.047***		0.102*	0.495
1st Tertile		Ref		Ref		
2st Tertile		0.522 (0.26; 1.04)	0.066	0.467 (0.2; 1.02)	0.055	
3st Tertile		1.217 (0.67; 1.04)	0.520	0.987 (0.50; 1.93)	0.971	
G_Mitsuokella	B		0.148*		0.426*	0.768
1st Tertile		Ref		Ref		
2st Tertile		1.013 (0.54; 1.90)	0.975	1.070 (0.51; 2.23)	0.857	
3st Tertile		1.662 (0.93; 3.01)	0.094	1.516 (0.76; 3.01)	0.235	
G_Veillonella	B		0.442*		0.299*	0.695
1st Tertile		Ref		Ref		
2st Tertile		0.740 (0.42; 1.30)	0.297	0.647 (0.34; 1.22)	0.177	
3st Tertile		0.712 (0.41; 1.25)	0.238	0.643 (0.34; 1.21)	0.170	
G_Fusobacterium	B		0.975*		0.960*	0.975
1st Tertile		Ref		Ref		
2st Tertile		1.079 (0.53; 2.21)	0.834	1.118 (0.51; 2.45)	0.780	
3st Tertile		1.079 (0.53; 2.21)	0.834	1.086 (0.49; 2.41)	0.840	
G_Victivallis	B		0.068*		0.196*	0.620
1st Tertile		Ref		Ref		
2st Tertile		1.555 (0.92; 2.62)	0.097	1.555 (0.87; 2.78)	0.132	
3st Tertile		1.814 (1.07; 3.07)	**0.027**	1.618 (0.90; 2.92)	0.109	
G_Parasutterella	B		0.101*		0.076*	0.495
1st Tertile		Ref		Ref		
2st Tertile		1.828 (1.04; 3.23)	**0.037**	2.077 (1.10; 3.92)	**0.024**	
3st Tertile		1.146 (0.67; 1.96)	0.616	1.514 (0.83; 2.76)	0.177	
G_Sutterella	B		**0.041***		0.230*	0.639
1st Tertile		Ref		Ref		
2st Tertile		1.092 (0.65; 1.82)	0.735	0.930 (0.52; 1.65)	0.804	
3st Tertile		1.916 (1.12; 3.28)	0.018	1.518 (0.83; 2.77)	0.173	
G_Bilophila	B		0.111*		0.234*	0.639
1st Tertile		Ref		Ref		
2st Tertile		1.921 (0.85; 4.36)	0.118	1.947 (0.79; 4.80)	0.148	
3st Tertile		0.825 (0.41; 1.64)	0.581	0.933 (0.43; 2.03)	0.863	
G_Desulfovibrio	B		**0.008***		**0.043***	0.495
1st Tertile		Ref		Ref		
2st Tertile		2.323 (1.27; 4.25)	**0.006**	2.229 (1.14; 4.35)	**0.019**	
3st Tertile		2.078 (1.15; 3.75)	**0.015**	1.850 (0.95; 3.61)	0.071	
G_Campylobacter	B		0.516*		0.415*	0.768
1st Tertile		Ref		Ref		
2st Tertile		0.708 (0.39; 1.27)	0.250	0.657 (0.35; 1.24)	0.194	
3st Tertile		0.853 (0.48; 1.51)	0.586	0.762 (0.41; 1.42)	0.391	
G_Succinivibrio	B		0.890*		0.827*	0.932
1st Tertile		Ref		Ref		
2st Tertile		1.009 (0.45; 2.28)	0.982	1.046 (0.41; 2.59)	0.941	
3st Tertile		1.186 (0.54; 2.61)	0.671	0.797 (0.32; 1.96)	0.621	
G_Escherichia/Shigella	B		0.928*		0.865*	0.957
1st Tertile		Ref		Ref		
2st Tertile		0.869 (0.43; 1.77)	0.698	1.130 (0.76; 2.47)	0.759	
3st Tertile		0.926 (0.45; 1.90)	0.834	0.912 (0.42; 1.99)	0.817	
G_Haemophilus	B		0.327*		0.640*	0.863
1st Tertile		Ref		Ref		

2st Tertile		1.445 (0.86; 2.42)	0.164	1.269 (0.71; 2.26)	0.418	
3st Tertile		1.054 (0.63; 1.75)	0.839	0.993 (0.56 1.75)	0.982	
G_Cloacibacillus	B		0.140*		0.052*	0.495
1st Tertile		Ref		Ref		
2st Tertile		0.534 (0.23; 1.26)	0.150	0.411 (0.15; 1.14)	0.089	
3st Tertile		1.234 (0.61; 2.53)	0.566	1.406 (0.63; 3.13)	0.403	
G_Akkersmansia	B		0.976*		0.934*	0.967
1st Tertile		Ref		Ref		
2st Tertile		1.023 (0.61; 1.73)	0.932	1.016 (0.57; 1.83)	0.957	
3st Tertile		1.062 (0.63; 1.80)	0.825	0.916 (0.51; 1.65)	0.770	
		**Phylum**				
P_Actinobacteria	Q		**0.025***		0.196*	0.784
1st tertile		Ref		Ref		
2st tertile		0.703 (-0.18; 0.32)	0.564	0.049 (-0.21; 0.31)	0.707	
3st tertile		0.330 (0.08; 0.58)	**0.010**	0.228 (-0.03; 0.49)	0.087	
P_Bacteroidetes	Q		0.555*		0.941*	0.941
1st tertile		Ref		Ref		
2st tertile		0.068 (-0.18; 0.32)	0.596	0.046 (-0.22; 0.31)	0.736	
3st tertile		0.139 (-0.11; 0.39)	0.278	0.034 (-0.23; 0.30)	0.804	
P_Firmicutes	Q		0.145*		0.792*	0.941
1st tertile		Ref		Ref		
2st tertile		-0.066 (-0.32; 0.18)	0.603	-0.036 (-0.30; 0.23)	0.790	
3st tertile		-0.243 (-0.49; 0.01)	0.057	-0.092 (-0.36; 0.17)	0.498	
P_Proteobacteria	Q		0.376*		0.414*	0.828
1st tertile		Ref		Ref		
2st tertile		-0.065 (-0.32; 0.19)	0.609	-0.006 (-0.28; 0.27)	0.965	
3st tertile		-0.177 (-0.43; 0.07)	0.167	-0.164 (-0.44; 0.11)	0.243	

# Model adjusted for perinatal variables (gestational age, type of delivery, birth weight, parity, total family income, sex, skin color), breastfeeding duration, other sources of consumption in the NOVA classification, BMI and use of antibiotics 6 months before the interview at age 12.

* P-value for the Test Parm

Q: Quantitative model (assessed using linear regression, with results expressed as beta coefficients); B: Binary model (assessed using binary logistic regression, with results expressed as Odds Ratios, OR).

Table S2 Prevalence of high ultra-processed food (UPF) consumption at 6, 11 and 12 years of age, according to covariates in a subsample of the Pelotas 2004 Birth Cohort.

Covariables	Ultra-processed (highest tercile)					
	6 years		11 years		12 years	
	N (%)	p-value*	N (%)	p-value*	N (%)	p-value*
**Perinatal and maternal characteristics**						
**Sex**		0.071		0.183		**0.015**
Male	69 (37.5%)		70 (36.5%)		75 (39.1%)	
Female	49 (28.5%)		52 (29.9%)		47 (27.0%)	
**Skin color**		0.288		**< 0.001**		0.073
White	79 (32.6%)		69 (27.8%)		74 (29.8%)	
Black	19 (45.2%)		26 (57.8%)		20 (44.4%)	
Brown	18 (28.1%)		26 (40.6%)		26 (40.6%)	
Others	2 (25.0%)		1(12.5%)		1 (12.5%)	
**Mother's age**		0.572		0.934		0.986
≤ 24	59 (34.7%)		60 (34.3%)		58 (33.1%)	
25-34	43 (30.1%)		48 (32.4%)		50 (33.8%)	
≥ 35	16 (37.2%)		14 (32.6%)		14 (32.6%)	
**Gestational age**		0.353		0.309		**0.007**
≤ 36 weeks	10 (41.7%)		11 (42.3%)		15 (57.7%)	
37-41 weeks	107 (32.4%)		110 (32.5%)		107 (31.7%)	
**Type of delivery**		0.497		**0.010**		0.139
Vaginal	64 (34.8%)		74 (39.6%)		69 (36.9%)	
Cesarean	54 (31.4%)		48 (26.8%)		53 (29.6%)	
**Birth weight**		0.498		0.622		0.556
< 2,500 g	8 (28.6%)		10 (32.3%)		13 (41.9%)	
2,500-3,499 g	78 (35.5%)		72 (31.7%)		73 (32.2%)	
> 3,500 g	32 (29.6%)		40 (37.0%)		36 (33.3%)	
**Duration of breastfeeding**		0.685		0.606		0.456
< 12 months	73 (33.0%)		79 (32.6%)		79 (34.8%)	
12-24 months	18 (29.0%)		20 (31.5%)		17 (26.6%)	
> 24 months	26 (32.1%)		28 (38.4%)		25 (34.2%)	
**Parity**		**0.010**		0.550		**0.036**
1	40 (26.3%)		49 (31.2%)		41 (26.1%)	
2-3	63 (41.7%)		56 (36.6%)		60 (39.2%)	
4 or more	14 (26.9%)		17 (30.9%)		21 (38.2%)	
**Household income (quintiles)**		0.259		**0.006**		**0.047**
1st quintile (lower)	25 (33.3%)		30 (37.5%)		35 (43.7%)	
2nd quintile	27 (38.0%)		31 (43.1%)		28 (38.9%)	
3rd quintile	23 (33.8%)		29 (42.0%)		22 (31.9%)	
4th quintile	28 (37.8%)		18 (23.4%)		22 (28.6%)	
5th quintile	15 (22.1%)		14 (20.6%)		15 (22.1%)	
**Adolescent characteristics**						
**Grams of in natura**		0.079		**< 0.001**		**< 0.001**
Less than or equal to median	65 (29.7%)		54 (21.9%)		43 (20.3%)	
Greater than median	53 (38.7%)		68 (56.7%)		79 (51.3%)	
**Grams of ingredients**		0.955		**< 0.001**		**0.022**
Less than or equal to median	75 (33.0%)		59 (26.5%)		66 (28.9%)	
Greater than median	43 (33.3%)		63 (44.1%)		56 (40.6%)	
**Grams of processed food**		0.325		**0.001**		**< 0.001**
Less than or equal to median	83 (34.9%)		64 (27.0%)		45 (23.7%)	
Greater than median	35 (29.7%)		58(45.0%)		77 (43.7%)	
**Nutritional status**		0.215		0.859		**0.050**
1st tertile (lower)	46 (39.7%)		40 (32.5%)		45 (37.2%)	
2nd tertile	36 (32.4%)		39 (32.2%)		46 (38.0%)	
3rd tertile	33 (28.9%)		43 (35.3%)		30 (24.8%)	

The highest tertile was used as the benchmark for the highest level of UPF consumption at each respective age.

* Fisher's exact test

N = Number of individuals with high UPF consumption and corresponding percentage
